# Accelerated brain aging towards transcriptional inversion in a zebrafish model of the K115fs mutation of human *PSEN2*

**DOI:** 10.1371/journal.pone.0227258

**Published:** 2020-01-24

**Authors:** Nhi Hin, Morgan Newman, Jan Kaslin, Alon M. Douek, Amanda Lumsden, Seyed Hani Moussavi Nik, Yang Dong, Xin-Fu Zhou, Noralyn B. Mañucat-Tan, Alastair Ludington, David L. Adelson, Stephen Pederson, Michael Lardelli

**Affiliations:** 1 Bioinformatics Hub, School of Biological Sciences, University of Adelaide, Adelaide, South Australia, Australia; 2 Alzheimer’s Disease Genetics Laboratory, School of Biological Sciences, University of Adelaide, Adelaide, South Australia, Australia; 3 Australian Regenerative Medicine Institute, Monash University, Clayton, Victoria, Australia; 4 College of Medicine and Public Health, and Centre for Neuroscience, Flinders University, Adelaide, South Australia, Australia; 5 School of Pharmacy and Medical Sciences, University of South Australia, Adelaide, South Australia, Australia; 6 Centre for Bioinformatics and Computational Genetics, School of Bioogical Sciences, Adelaide, South Australia, Australia; Icahn School of Medicine at Mount Sinai, UNITED STATES

## Abstract

**Background:**

The molecular changes involved in Alzheimer’s disease (AD) progression remain unclear since we cannot easily access antemortem human brains. Some non-mammalian vertebrates such as the zebrafish preserve AD-relevant transcript isoforms of the *PRESENILIN* genes lost from mice and rats. One example is PS2V, the alternative transcript isoform of the *PSEN2* gene. PS2V is induced by hypoxia/oxidative stress and shows increased expression in late onset, sporadic AD brains. A unique, early onset familial AD mutation of *PSEN2*, K115fs, mimics the PS2V coding sequence suggesting that forced, early expression of PS2V-like isoforms may contribute to AD pathogenesis. Here we use zebrafish to model the K115fs mutation to investigate the effects of forced PS2V-like expression on the transcriptomes of young adult and aged adult brains.

**Methods:**

We edited the zebrafish genome to model the K115fs mutation. To explore its effects at the molecular level, we analysed the brain transcriptome and proteome of young (6-month-old) and aged (24-month-old) wild type and heterozygous mutant female sibling zebrafish. Finally, we used gene co-expression network analysis (WGCNA) to compare molecular changes in the brains of these fish to human AD.

**Results:**

Young heterozygous mutant fish show transcriptional changes suggesting accelerated brain aging and increased glucocorticoid signalling. These early changes precede a transcriptional ‘inversion’ that leads to glucocorticoid resistance and other likely pathological changes in aged heterozygous mutant fish. Notably, microglia-associated immune responses regulated by the ETS transcription factor family are altered in both our zebrafish mutant model and in human AD. The molecular changes we observe in aged heterozygous mutant fish occur without obvious histopathology and possibly in the absence of Aβ.

**Conclusions:**

Our results suggest that forced expression of a PS2V-like isoform contributes to immune and stress responses favouring AD pathogenesis. This highlights the value of our zebrafish genetic model for exploring molecular mechanisms involved in AD pathogenesis.

## Introduction

Alzheimer’s disease (AD) is the leading cause of dementia, a condition characterised by the progressive decline of memory and cognition. Like other neurodegenerative diseases, AD affects diverse cellular processes in the brain, including mitochondrial function [1, 2], metal ion homeostasis [3–5], lipid metabolism [6–8], immune responses [9, 10], synaptic transmission [11], and protein folding and trafficking [12, 13]. Dysregulation of these processes eventually results in severe atrophy of several brain regions (reviewed by Braak and Braak [14] and Masters et al. [15]). Consequently, late stages of AD are likely to be much more difficult to treat than earlier stages of AD, contributing to our failure to discover ameliorative drugs [16].

The pathological processes that result in AD are likely to initiate decades before clinical symptoms arise. Decreased levels of soluble amyloid beta (Aβ) peptides in the cerebrospinal fluid is one of the earliest markers of both sporadic and familial forms of AD, preceding disease onset by 20–30 years [17, 18], while vascular changes are likely to occur even earlier [19]. Individuals possessing highly penetrant, dominant mutations in genes linked to the familial form of AD (fAD) such as *PSEN1* show structural and functional changes in their brains as early as 9 years of age, despite being cognitively normal [20, 21]. Similar findings are evident in young adults carrying the ε4 allele of *APOE*, the major risk gene for the sporadic form of AD [22]. To prevent AD, we must identify the stresses underlying these early pathological changes. However, detailed molecular analysis of the brains of asymptomatic young adult fAD mutation carriers is currently impossible.

Analysing high-throughput ‘omics data (e.g. transcriptomic, proteomic) is a comprehensive and relatively unbiased approach for studying complex diseases like AD. Over the past decade, numerous post-mortem AD brains have been profiled using microarray and RNA-seq technologies, exposing an incredibly complex and interconnected network of cellular processes implicated in the disease [23, 24]. Unfortunately, analysing post-mortem AD brains does not discern which cellular processes are responsible for initiating the cascade of events leading to AD.

Animal models can assist exploration of the early molecular changes that promote AD. However, early “knock-in” mouse models that attempted to model the genetic state of human fAD showed no obvious histopathology [25–27]. Modern ‘omics technologies provide molecular-level descriptions of disease states, but these technologies were not available when the early knock-in models were made. Subsequent transgenic models of AD constructed with multiple genes and/or mutations have displayed what are assumed to be AD-related histopathologies and these have also been analysed by ‘omics methods. However recent analysis of brain transcriptomes from five different transgenic AD models showed little concordance with human, late onset, sporadic AD brain transcriptomes. Worse still, none of the models were concordant with each other [28].

The overwhelming majority of fAD mutations are present in a heterozygous state in human patients. Despite this, there has been a lack of detailed molecular investigation of the young adult brains of any animal model closely imitating the human fAD genetic state–i.e. heterozygous for a fAD-like mutation in a single, endogenous gene. Previously, we used zebrafish to analyse the unique, frameshifting fAD mutation of human *PRESENILIN2* (*PSEN2*), K115fs, that inappropriately mimics expression of a hypoxia-induced truncated isoform of PSEN2 protein, PS2V [29–32]. Mice and rats have lost the ability to express PS2V [33] (and the fAD genes of these rodents are evolving more rapidly than in many other mammals [33]), but in zebrafish, this isoform is expressed from the animal’s *psen1* gene [32]. Consequently, to model and explore early changes in the brain contributing to AD pathogenesis, we have now used gene-editing technology to introduce a K115fs-equivalent mutation into the zebrafish *psen1* gene, K97fs. In this paper, we analyse data collected from young adult (6-month-old) and aged (24-month-old) adult heterozygous mutant and wild type zebrafish brains to comprehensively assess gene and protein expression changes in the brain due to aging and this mutation. At the molecular level, we find that the young heterozygous mutant brains show elements of accelerated aging while aged heterozygous mutant brains appear to ‘invert’ into a distinct, and presumably pathological, state. Our results highlight the important role that non-transgenic models of fAD mutations in a heterozygous state play in elucidating mechanisms of AD pathogenesis.

## Results

**Fig 1:**
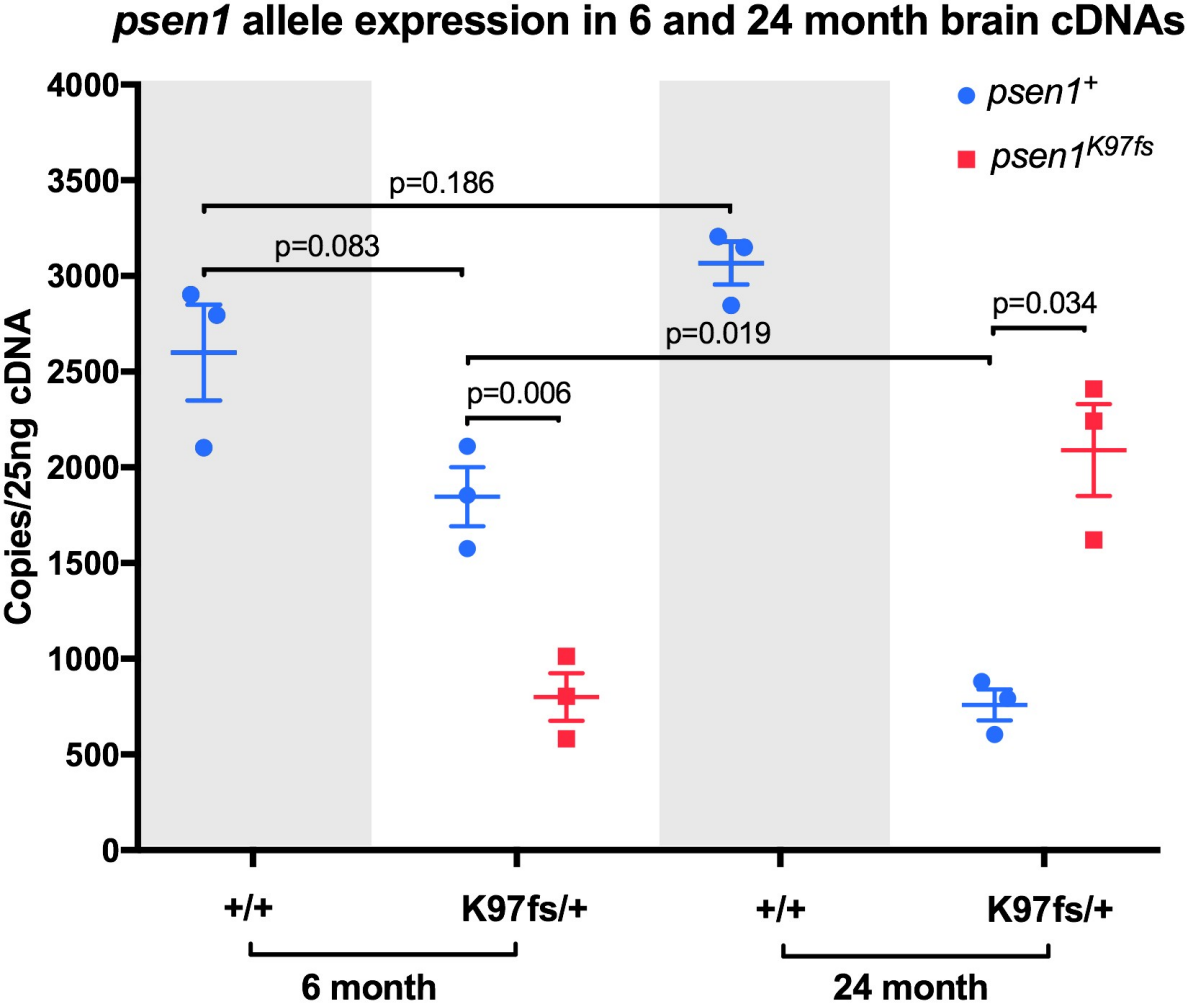
Quantification of heterozygous mutant and wild type allele relative transcript expression. Digital quantitative PCRs specifically detecting transcripts from the heterozygous mutant (K97fs) or wild type (+) alleles of *psen1* were performed using cDNA synthesised from total brain mRNA from fish at 6 and 24 months of age. Means and standard error of the means are indicated, and *p*-values are from two-sample *t*-tests assuming unequal variances.

Gene editing in zebrafish to produce the *psen1* K97fs mutation is described in the **Materials and Methods** and in **Fig A** in S1 File. To confirm that the K97fs mutation of *psen1* forces measurable expression of a PS2V-like transcript under normoxic conditions we performed digital quantitative PCR (dqPCR) specifically detecting either heterozygous mutant or wild type transcript sequences in cDNA synthesised from the brains of female 6-month-old (young) and 24-month-old (aged) *psen1*^K97fs/+^ (heterozygous mutant) and *psen1*^+/+^ (wild type) zebrafish (Fig 1). We only included female fish to reduce variability between samples and minimise confounding by potential gender-specific gene expression patterns, given that females are more vulnerable to AD and that gender-specific changes have been documented in AD [34, 35]. K97fs transcripts constitute approximately 30% of the *psen1* transcripts detected in young brains and over 70% of the detected transcripts in aged brains. Despite these different biases in heterozygous mutant and wild type transcript expression, the total levels of *psen1* transcript appeared similar between heterozygous mutant and wild type fish at either age. This supports that the K97fs mutant transcript (like PS2V transcripts in humans) is not completely degraded by nonsense mediated decay despite possession of a premature termination codon [29]. PCR tests on cDNA from heterozygous mutant brains did not detect aberrant splicing of the *psen1* gene due to the K97fs mutation. We currently have no explanation for the observed bias, or its age-dependent change, between the expression of the heterozygous mutant versus wild type *psen1* transcripts. The extent of the decrease in the wild type *psen1* transcript in the aged heterozygous mutant brains means that this may contribute to any molecular phenotype caused by heterozygosity for the K97fs mutation in addition to the effects of the PS2V-like transcripts.

**Fig 2:**
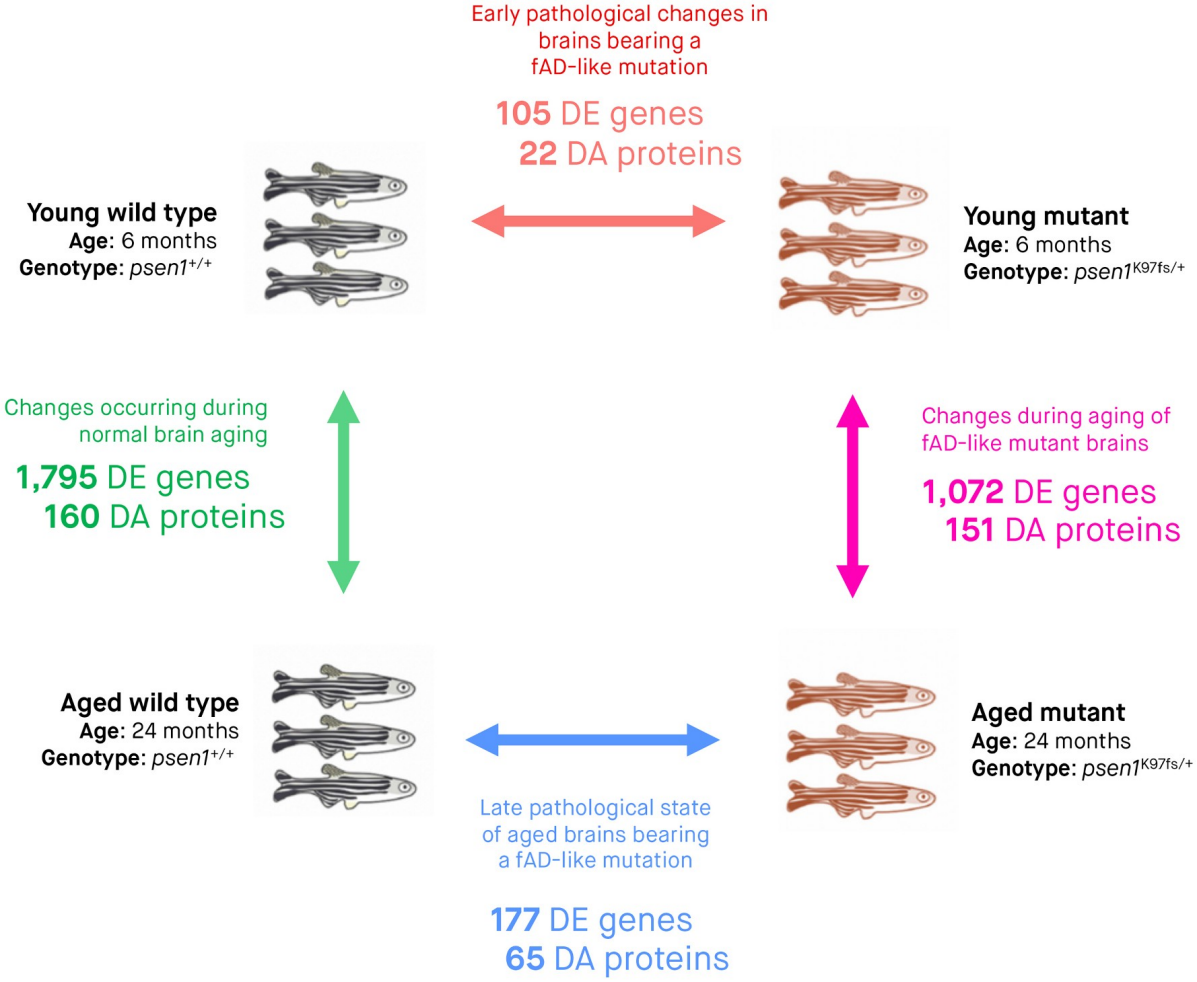
Summary of experimental groups, differentially expressed (DE) genes and differentially abundant (DA) proteins. Three biological replicates (entire zebrafish brains) were subjected to RNA-seq and LC-MS/MS for each of the four experimental conditions. Arrows indicate pairwise comparisons (to identify DE genes and DA proteins) between experimental conditions. The numbers of DE genes and DA proteins determined from RNA-seq and LC-MS/MS analyses are indicated underneath the arrow for each comparison. We considered genes to be DE and proteins to be DA if the False Discovery Rate [FDR]-adjusted *p*-value of their moderated *t*-test (*limma*) was below 0.05. All zebrafish of the same age are siblings raised in the same tank.

To determine whether the K97fs mutation in the zebrafish *psen1* gene induces changes in the expression of other genes and proteins, we removed entire brains of heterozygous mutant and wild type adult zebrafish for total RNA sequencing (RNA-seq) and label-free tandem mass spectroscopy (LC-MS/MS) when zebrafish were 6 months (young adult) and 24 months (aged adult) old. We used three biological replicates to represent each of the four experimental conditions (young wild type, young heterozygous mutant, aged wild type, aged heterozygous mutant), and performed pairwise comparisons between experimental conditions to determine differentially expressed (DE) genes and differentially abundant (DA) proteins (Fig 2). Full lists of DE genes and DA proteins are provided in S1 and S2 Tables.

### Gene expression changes in the heterozygous mutant zebrafish reveal accelerated brain aging followed by inversion into a presumably pathological state

**Fig 3:**
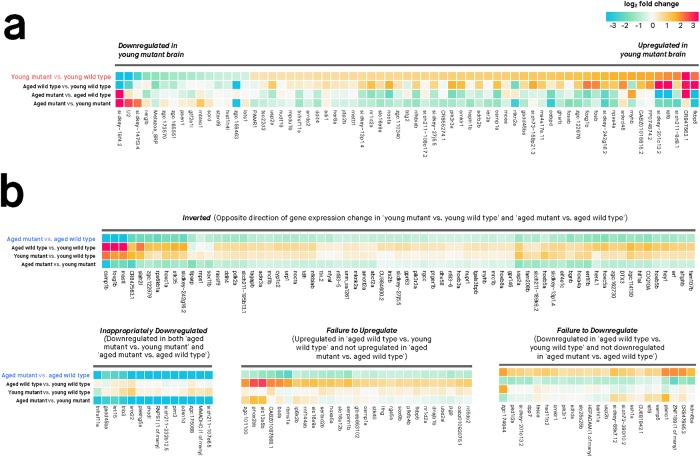
Differential gene expression between heterozygous mutant (*psen1*^*K97fs/+*^) and wild type (*psen1*^+/+^) zebrafish brains at 6 months (young) and 24 months (aged). Only genes with absolute log_2_ fold change > 0.5 are shown. Genes were considered differentially expressed if their moderated *t*-test FDR-adjusted *p*-value was below 0.05. **(A) Differentially expressed genes at 6 months. (B) Differentially expressed genes at 24 months**. The differentially expressed genes are grouped into clusters based on gene expression changes across the four comparisons. Overall, note the similar expression changes in ‘young heterozygous mutant vs. young wild type’ and ‘aged wild-type vs. young wild-type’ and the contrast of these to comparisons involving aged heterozygous mutants. This illustrates the accelerated brain aging in young heterozygous mutant brains and the "inverted" gene expression pattern of aged heterozygous mutant brains.

The brains of children or young adults carrying fAD mutations display morphological and functional differences compared to age-matched individuals without these mutations [20, 21]. Consequently, we hypothesised that gene expression in the brains of young adult (6-month-old) zebrafish carrying this K115fs-like mutation would also be altered when compared to wild type zebrafish siblings. Overall, we find supporting evidence for 105 genes that are differentially expressed in young heterozygous mutant brains relative to wild type brains (65 up-regulated, 40 down-regulated; FDR-adjusted *p*-value < 0.05) (**Fig B in** S1 File). Of these 105 genes, 65 have an estimated log_2_ fold change greater than 0.5 (or less than -0.5) in the ‘young heterozygous mutant vs. young wild type’ comparison (Fig 3A). By examining the expression of these genes in the other three comparisons described in Fig 2, we observe two important phenomena:

1. **Accelerated aging genes are associated with increased immune response**: 62% (65/105) of the genes that are DE in 6-month-old heterozygous mutant brains (‘young heterozygous mutant vs. young wild type’) show the same direction of expression change during normal aging (‘aged wild type vs. young wild type’). However, far more genes are DE during normal aging (1,795 compared to 105). This suggests that the 6-month-old heterozygous mutant brains may demonstrate accelerated aging for a subset of cellular functions. As an initial step to explore these altered cellular functions, we applied functional enrichment analysis on these 65 genes and discovered significant enrichment in an MSigDB gene set relating to immune response genes that are up-regulated following lipopolysaccharide treatment “GSE9988 LPS VS VEHICLE TREATED MONOCYTE UP” (Bonferroni adjusted *p*-value 0.000948) (S3 Table).
2. **Age-dependent ‘inversion’ pattern**: A subset of 63 genes with increased expression in 6-month-old heterozygous mutant brains (‘young heterozygous mutant vs. young wild type’) show decreased expression in 24-month-old heterozygous mutant brains (‘aged heterozygous mutant vs. aged wild type’). We call this expression pattern an age-dependent ‘inversion’ between heterozygous mutant and wild type brains, and explore the biological relevance of the genes involved in this inversion pattern later.

By comparing gene expression in 24-month-old heterozygous mutant and wild type zebrafish brains, we can gain insight into a putatively pathological transcriptomic state present in the brains of aged zebrafish carrying this mutation. We find supporting evidence for 177 genes that are differentially expressed in heterozygous mutant brains relative to wild type brains (139 down-regulated, 38 up-regulated; FDR-adjusted *p*-value < 0.05) (Fig 3B; **Fig B in** S1 File). Note that not all of these genes are shown in Fig 3B, which only includes genes with log_2_ fold change values greater than 0.5 or less than -0.5. To allow for easier interpretation of these 177 genes, we used hierarchical clustering to separate them into groups with distinct expression patterns based on all four brain-types:

- **Inverted (63 genes)**: Defined as genes showing opposite fold-changes in young heterozygous mutant brains (‘young heterozygous mutant vs. young wild type’) compared to aged heterozygous mutant brains (‘aged heterozygous mutant vs. aged wild type’). To be included in this group, genes were required to have an FDR-adjusted *p*-value < 0.05 in either the ‘young heterozygous mutant vs. young wild type’ or ‘aged heterozygous mutant vs. aged wild type’ comparison and an unadjusted *p*-value < 0.05 in the other comparison.
- **Inappropriately down-regulated (57 genes)**: Defined as genes that are down-regulated in the ‘aged heterozygous mutant vs. young heterozygous mutant’ and ‘aged heterozygous mutant vs. aged wild type’ comparisons (FDR-adjusted *p*-value < 0.05 in both).
- **Failure to up-regulate (94 genes)**: Defined as genes that are up-regulated during normal aging (FDR-adjusted *p*-value < 0.05 in the ‘aged wild type vs. young wild type’ comparison) but not up-regulated in the ‘aged heterozygous mutant vs. aged wild type’ comparison.
- **Failure to down-regulate (26 genes)**: Defined as genes that are down-regulated during normal aging (FDR-adjusted *p*-value < 0.05 in the ‘aged wild type vs. young wild type’ comparison) but not down-regulated in the ‘aged heterozygous mutant vs. aged wild type’ comparison.

To determine whether these different component groups of the gene expression patterns are biologically relevant, we assessed each group’s functional enrichment using Gene Ontology terms, MSigDB gene sets, and Reactome and Interpro pathways (summarised in S3 Table; full results in S4 Table. Overall, we find statistically significant enrichment (Bonferroni adjusted *p*-value < 0.05) for all groups except for the ‘failure to down-regulate’ group. The ‘inverted’ group is significantly enriched in several gene sets related to stress and immune response; the ‘inappropriately down-regulated’ group is significantly enriched in developmental transcription factors including homeobox genes; the ‘failure to up-regulate’ group is significantly enriched in immune responses.

### Gene expression changes during aging of the heterozygous mutant zebrafish brains partially overlap with normal brain aging

Gene expression changes associated with aging in the wild type and heterozygous mutant zebrafish brains only partially overlap. When comparing 24-month-old and 6-month-old wild type zebrafish brains, 1,795 genes show differential expression. However, when comparing 24-month-old and 6-month-old heterozygous mutant zebrafish brains, 1,072 genes show altered expression (FDR-adjusted *p*-value < 0.05). When comparing these two sets of genes, only 525 genes show fold-changes in the same direction during wild type and heterozygous mutant aging. These genes can be considered an ‘aging signature’ and are functionally enriched in gene ontology terms relating to immune function (S3 Table). This suggests that the heterozygous mutant fish still preserve some immune-related gene expression changes that occur during normal aging.

### Gene expression changes are likely not due to changes in proportions of brain cell types

It is possible that changes in the proportions of different cell types in the brain could result in genes being falsely interpreted as differentially expressed. As a preliminary test to see whether our observations of differential gene expression were artefacts of change in the proportions of major brain cell types (e.g. astrocytes, microglia, neurons, oligodendrocytes), we checked for noticeable changes in the average expression for sets of marker genes characteristic of each of the major brain cell types across the samples in each experimental condition (young wild type, young heterozygous mutant, aged wild type, aged heterozygous mutant). Representative marker genes for microglia were obtained from Oosterhof et al. [36] while gene markers for astrocytes, neurons, and oligodendrocytes were obtained from Lein et al. [37] The number of genes used to calculate the average gene expression (in logCPM) was 41 (astrocyte), 99 (microglia), 77 (neuron) and 78 (oligodendrocyte). Although this method is limited in that it does not account for the significant diversity within these broader cell types nor regional brain differences, this level of analysis suggests that broadly, the average expression of gene markers for the major neural cell types does not appear to change much across experimental conditions. In addition, no obvious outlier samples were evident (**Fig C in** S1 File).

### Regulation of gene expression in the heterozygous mutant zebrafish brains differs from normal brain aging

A transcription factor can regulate gene expression by binding to a specific DNA motif in the promoter region of a gene. We hypothesised that changes in gene expression during normal aging or differences in gene expression between heterozygous mutant and wild type brains could be driven by differences in transcription factor activity. To test this, we examined gene promoter regions for enriched motifs corresponding to known transcription factor binding sites (summarised in S5 Table; full results in S6 Table). Overall, we find:

1. **Numerous known transcription factors likely drive the gene expression changes that occur during normal zebrafish brain aging**. As wild type brains age, the genes which are differentially expressed are significantly enriched in many known motifs. These motifs correspond to binding sites for interferon regulatory factors (e.g. IRF1, IRF2, IRF8); a binding site for the PU.1-IRF8 complex; an interferon-stimulated response element (ISRE); and binding sites for various transcription factors important for essential cellular processes like proliferation, differentiation, and apoptosis (Atf3, Fra2, Ets-distal, AP-1, Fra1, JunB, BATF, and ZNF264).
2. **Altered glucocorticoid signalling in heterozygous mutant zebrafish brains is likely to contribute to a pathological state**. Promoters of genes that are differentially expressed in the ‘aged heterozygous mutant vs. aged wild type’ comparison are significantly enriched in the glucocorticoid receptor element motif (GRE) (Bonferroni *p*-value = 0.0057). Interestingly, the subset of genes showing inappropriate downregulation (down-regulated in the ‘aged heterozygous mutant vs. young heterozygous mutant’ and ‘aged heterozygous mutant vs. aged wild type’ comparisons) is more strongly enriched again in the GRE motif (Bonferroni *p*-value = 0.0001), suggesting that genes that are normally activated by glucocorticoid signalling during aging may not be activated in aged heterozygous mutant brains. This altered glucocorticoid signalling appears to be present even in young zebrafish brains, as genes showing inverted behaviour (opposite direction of differential expression in ‘young heterozygous mutant vs. young wild type’ and ‘aged heterozygous mutant vs. aged wild type’ comparisons) are also enriched in the GRE motif (Bonferroni *p*-value = 0.0047). Because these inverted genes tend to show high expression in young heterozygous mutant brains (i.e. up-regulated in the ‘young heterozygous mutant vs. young wild type’ comparison) and low expression in aged heterozygous mutant brains (i.e. down-regulated in the ‘aged heterozygous mutant vs. aged wild type’ comparison), this suggests that young heterozygous mutant zebrafish brains may initially exhibit abnormally increased glucocorticoid signalling, while aged heterozygous mutant brains later exhibit abnormally decreased glucocorticoid signalling. Notably, the inverted genes containing a GRE motif in their promoters include *COQ10A* (encodes Coenzyme Q10, a key component of the electron transport chain and free-radical scavenging antioxidant); *pik3r3a* (encodes regulatory subunit gamma of phosphoinositide 3-kinase, an enzyme that interacts with insulin growth factor 1 receptor among other proteins); *mmadhc* (encodes a protein involved in an early and essential step of vitamin B12 metabolism), *plk3* (polo-like kinase 3, involved in stress response and double-stranded DNA repair), and *fkbp5* (encodes FK506 binding protein, involved in regulating immune and stress responses, protein trafficking and folding, and glucocorticoid receptor regulation). A list of zebrafish genes containing the GRE promoter motif is provided in S7 Table.

### Gene expression changes in the heterozygous mutant zebrafish indicate vast changes to cellular processes and pathways

**Fig 4:**
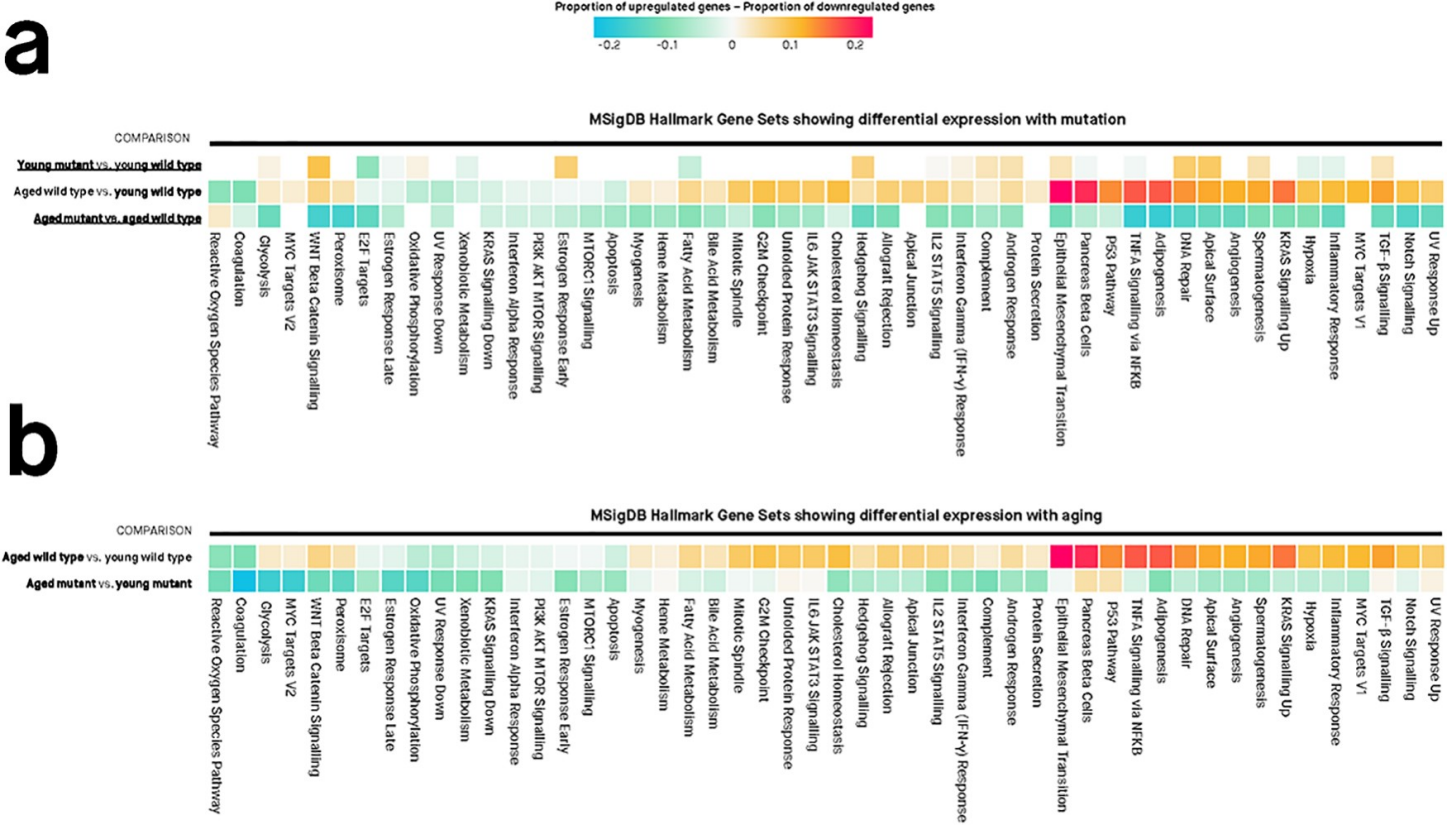
Differential gene set expression in heterozygous mutant (*psen1*^K97fs/+^) zebrafish brains compared to wild type siblings. Values in each cell are the estimated proportions of up- and down-regulated genes for each gene set, for any particular pairwise comparison shown to the left of the cells. A missing cell indicates that the particular gene set is not differentially expressed for that particular pairwise comparison. Colours of cells are proportional to the difference between the proportion of up- and down-regulated genes in a gene set. Differentially expressed gene sets have Mixed FDR below 0.05, indicating genes within the gene set show statistically significantly altered (up and/or down) expression for a particular comparison. The genes in each gene set are defined using the “Hallmark” gene set collection at the Molecular Signatures Database (MSigDB). **(A) Gene sets showing differential expression between heterozygous mutant (*psen1***^***K97fs/+***^**) and wild type (*psen1***^**+/+**^**) zebrafish brains at 6 months (young) and 24 months (aged)**. The comparison representing normal aging (aged wild type vs. young wild type) is also shown to highlight the ‘accelerated aging’ phenomenon in the young heterozygous mutants. **(B) Gene sets showing differential expression during normal aging**. The aged K97fs/+ vs. young K97fs/+) comparison is also shown to highlight the phenomenon of aberrant aging in the heterozygous mutants.

A gene set is a group of genes that contribute to a known biological function, pathway, or state. A gene set test is an analysis used to evaluate whether a particular gene set is differentially expressed for a particular comparison. We used the FRY method to test whether ‘Hallmark’ gene sets from the Molecular Signatures Database, MSigDB [38] were associated with differential expression in each of the four comparisons (Fig 4 and S8 Table). Using an FDR-adjusted *p*-value < 0.05 to define a gene set as differentially expressed, we find:

1. **50 gene sets are differentially expressed during normal brain aging (‘aged wild type vs. young wild type’)** (middle row of heatmap, Fig 4A). This supports that many biological functions and pathways are altered during normal aging. For some gene sets, the proportion of genes that are up-regulated and down-regulated is similar (e.g. interferon alpha response, E2F targets, early estrogen response). However, other gene sets contain a predominance of up-regulated genes (e.g. epithelial mesenchymal transition, TNFA signalling via NFKB) or down-regulated genes (e.g. coagulation, reactive oxygen species pathway).
2. **22 gene sets are differentially expressed in young heterozygous mutant brains (‘young heterozygous mutant vs. young wild type’)** (top row of heatmap, Fig 4A). These 22 gene sets may represent earlier functional changes in the brain that occur due to this mutation. The gene sets implicate diverse processes including Wnt/β-catenin signalling, early estrogen response, DNA repair, hedgehog signalling and fatty acid metabolism. Similar to the pattern of accelerated aging observed in Fig 3, we also observe that most of the gene sets up-regulated in young heterozygous mutant brains are regulated in the same direction during normal aging. This is consistent with the idea that the biological changes in young heterozygous mutant brains may partially recapitulate those that occur during normal brain aging.
3. **44 gene sets are differentially expressed between aged heterozygous mutant and aged wild type brains** (bottom row, Fig 4A). These differentially expressed gene sets may represent the pathological state of aged zebrafish brains bearing this mutation. Importantly, 21 of the 22 gene sets that were differentially expressed in young heterozygous mutant brains (‘young heterozygous mutant vs. young wild type’) remain altered also when these are aged (‘aged heterozygous mutant vs. aged wild type’). However, the proportions of up- and down-regulated genes tend to differ; notably, several gene sets containing a predominance of up-regulated genes in the young heterozygous mutant brains contain a predominance of down-regulated genes in the old heterozygous mutant brains. These ‘inverted’ gene sets include biological functions and pathways as diverse as Wnt/β-catenin signalling, early estrogen response, hedgehog signalling, androgen response, epithelial mesenchymal transition, DNA repair, apical surface, and TGF-β signalling.
4. **Aging in the heterozygous mutant brains is similar but distinct from aging in wild type brains**. The 50 gene sets differentially expressed during normal brain aging are also differentially expressed during heterozygous mutant brain aging (‘aged heterozygous mutant vs. young heterozygous mutant’) (Fig 4B). However, proportions of up- and down-regulated genes differ from those in normal brain aging. This suggests that zebrafish brains bearing this mutation may not properly regulate certain gene sets during aging (e.g. cholesterol homeostasis, adipogenesis, DNA repair, hypoxia, Wnt/β-catenin signalling).

### Altered protein abundance in the heterozygous mutant zebrafish brains

**Fig 5:**
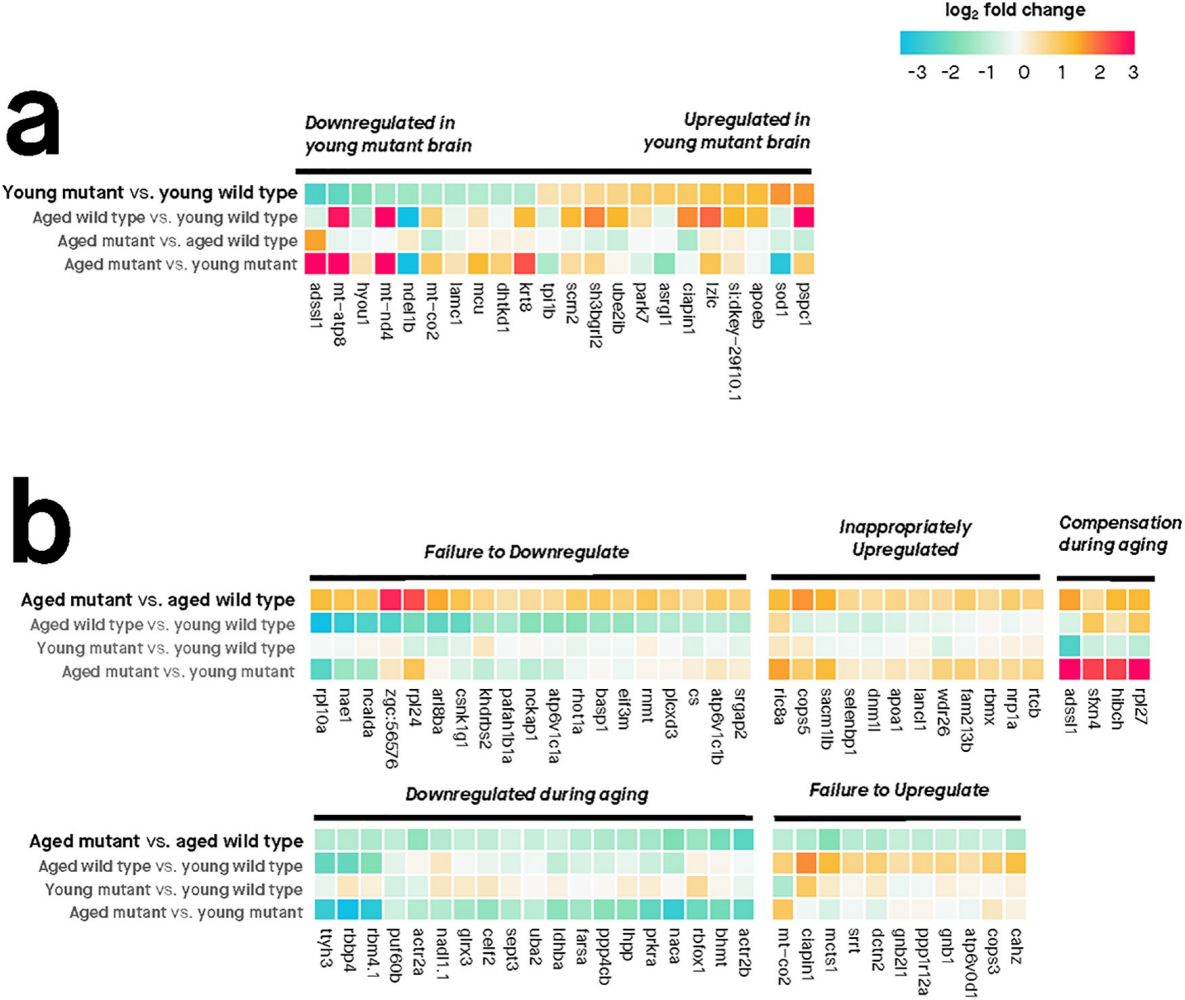
Protein abundance changes in the brains of heterozygous mutant (*psen1*^*K97fs/+*^) zebrafish compared to wild type (*psen1*^+/+^) siblings at 6 months (young) and 24 months (aged). Protein abundance was quantified at the peptide-level with LC-MS/MS (liquid chromatography tandem mass spectrometry) and differential abundance was assessed using moderated *t*-tests (*limma*). Differentially abundant proteins are defined as those with FDR-adjusted *p*-value < 0.05. Protein names were used to retrieve equivalent gene symbols for display purposes on these heatmaps. **(A) Differentially abundant proteins between young heterozygous mutant and wild type zebrafish brains. (B) Differentially abundant proteins between aged heterozygous mutant and wild type zebrafish brains**. The proteins have been clustered according to their abundance changes across the four comparisons.

Despite its high sensitivity, estimating gene expression does not capture regulatory processes or post-transcriptional modifications that might affect the amount of active protein. Correlation between gene expression and protein abundance in samples from multicellular organisms has been notoriously low [39], but analysing proteomics data alongside gene expression data has been shown to be an effective complementary approach [40]. Because of this, we decided to use LC-MS/MS to compare protein abundance in heterozygous mutant zebrafish brains relative to wild type siblings. Overall, 323 proteins were reliably quantified across all samples aged 6 or 24 months. Testing for differential protein abundance was done analogously to testing for differential gene expression, with differences at FDR-adjusted *p*-value < 0.05 considered statistically significant. 22 proteins were differentially abundant between 6-month-old heterozygous mutant and wild type brains, while 65 proteins were differentially abundant between 24-month-old heterozygous mutant and wild type brains (Fig 5; **Fig D in** S1 File). Unexpectedly, three proteins found to be differentially abundant between 6-month-old heterozygous mutant and wild type zebrafish have causative roles in human neurodegenerative diseases: apolipoprotein Eb (encoded by the zebrafish *apoeb* gene, orthologous to the major human genetic risk factor for sporadic AD, *APOE*), superoxide dismutase (encoded by the zebrafish *sod1* gene, orthologous to the human *SOD1* gene mutated in familial amyotrophic lateral sclerosis), and protein DJ-1 (encoded by the zebrafish *park7* gene, orthologous to the human *PARK7* gene mutated in familial Parkinson’s disease). Overall, correlation between gene expression and protein abundance was low with *r*_*s*_ = 0.4 at 6 months of age and *r*_s_ = 0.28 at 24 months of age (**Figs E** and **F** in S1 File). However, this is overall consistent with previously reported correlation coefficients in multicellular organisms that range from 0.09 to 0.68 [39]).

### Gene expression changes in the heterozygous mutant zebrafish brains can be compared to those in human AD

Our results indicate that gene expression changes involving diverse cellular processes occur in aged heterozygous mutant zebrafish brains. The K115fs mutation is a human fAD mutation, but the majority of human AD cases are sporadic, arise from diverse environmental and genetic risk factors, and can involve heterogenous pathological changes in the brain. Nevertheless, it may be informative to explore the extent to which the changes in aged heterozygous mutant zebrafish can model those in human AD.

To assess the similarity of these changes to human brains with AD, we compared gene expression patterns in our zebrafish RNA-seq dataset and an independent human RNA-seq dataset from the Mayo RNA-seq study. The Mayo RNA-seq dataset includes not only patients with AD (defined as having dementia symptoms, Braak neurofibrillary tangle stage IV or greater, and presence of amyloid pathology) and similarly aged controls, but also patients with other brain afflictions that recapitulate aspects of AD (“pathological aging” patients possessing amyloid pathology without dementia symptoms, and progressive supranuclear palsy patients possessing neurofibrillary tangle pathology but no amyloid pathology) [41].

**Fig 6:**
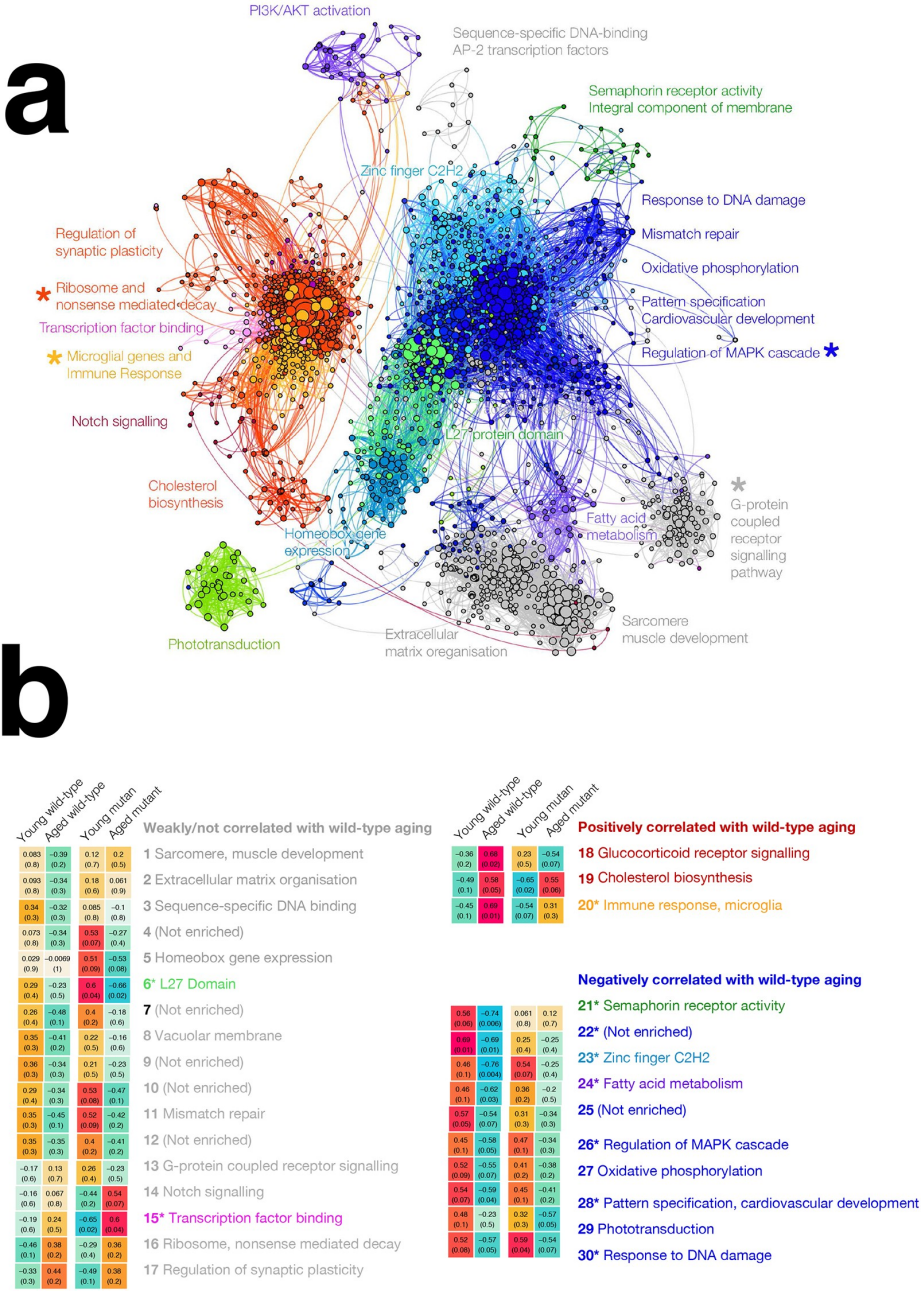
Zebrafish brain gene co-expression network. **(A) Gene co-expression network visualisation**. Each node represents one gene, with node size proportional to the number of connected nodes (co-expressed genes). Edges represent co-expression between two genes, with edge weight proportional to the strength of co-expression. The co-expression network is a signed adjacency matrix constructed from RNA-seq data from wild type and heterozygous mutant zebrafish brains at 6 and 24 months of age. Only nodes with at least four connections are shown. Gene "modules" are groups of genes with similar expression patterns across heterozygous mutant and wild type zebrafish brains. In this network, 30 gene modules were identified using a hierarchical clustering and branch cutting method. Modules showing no significant changes in expression are coloured grey, modules showing significantly increased expression during wild-type brain aging are coloured red, while modules showing significantly decreased expression during wild-type brain aging are coloured blue. Modules with other colours also show signficantly altered expression during heterozygous mutant brain aging. See **B** for details. Asterisks indicate zebrafish brain gene modules which are significantly preserved in a co-expression network constructed from an independent human brain dataset. **(B) Gene expression patterns of modules in the gene co-expression network across heterozygous mutant and wild type zebrafish brains at 6 months and 24 months of age**. Values shown in cells are hybrid Pearson-robust correlations between the overall gene expression in a module (summarised using the first principal component) and experimental condition encoded as a binary variable (6-month-old heterozygous mutant, 24-month-old heterozygous mutant, 6-month-old wild type, 24-month-old wild type). Values in parentheses are unadjusted Student correlation p-values. Modules showing potentially altered expression patterns during heterozygous mutant aging compared to wild-type aging are labelled with coloured text, with colours corresponding to module colours in **(A)**. Asterisks indicate zebrafish brain gene modules which are significantly preserved in a co-expression network constructed from an independent human brain dataset.

We constructed separate gene co-expression networks from the zebrafish and human RNA-seq datasets. Each network only included genes that were orthologs in humans and zebrafish. Whilst there are many methods for constructing a co-expression network of gene expression [42], we used the weighted gene co-expression network analysis (WGCNA) method [43], which has previously been used to group genes expressed in the brain into “modules” associated with biological functions or activities [44–48]. The zebrafish brain co-expression network is shown in Fig 6, and the human brain co-expression network is provided in **Fig G in** S1 File.

We identified 30 modules (i.e. groupings of genes) in the zebrafish brain co-expression network containing between 54 and 1221 genes each and 27 modules in the human brain co-expression network containing between 62 and 921 genes each. We used two methods to confirm that most modules represented functional relationships between genes: enrichment analysis (for identifying enriched biological functions and enriched promoter motifs), and correlating modules with particular traits of interest (age and/or *psen1* genotype). By correlating modules with particular zebrafish traits (age and *psen1* genotype), we identified 13 (out of 30) modules showing evidence of altered expression patterns in heterozygous mutant zebrafish brains (Fig 6B). Using enrichment analysis, we identified the biological relevance of each module in the zebrafish co-expression network (see Table 1 for a summary, and full enrichment analysis results are shown in S9–S11 Tables). Overall, the majority of modules in the zebrafish and human networks show significant enrichment in known functional annotations (e.g. Gene ontology terms, MSigDB gene sets, KEGG pathways, with Bonferroni-adjusted *p-*value < 0.05), supporting the idea that these modules are likely to represent biologically relevant groupings of genes. Some of the biological functions represented by different modules in the zebrafish brain include: G-protein coupled receptor signalling pathway (represented by module **13**), TGF-β and Wnt/β-catenin signaling (represented by module **15**), PI3K/AKT activation (represented by module **18**), immune response (represented by module **20**), regulation of MAPK cascade (represented by module **26**), and oxidative phosphorylation (represented by module **27**). The genes in several modules in the zebrafish network were also significantly enriched in promoter motifs including the glucocorticoid receptor element (GRE) motif (for genes in module **18**), GATA3 motif (for genes in module **23**), and numerous ETS transcription factor motifs (for genes in module **20**) (Bonferroni-adjusted *p*-values < 0.05) (S12 Table).

**Table 1:** Summary of modules in a co-expression gene expression network constructed from zebrafish RNA-seq data and their preservation in an independent human brain microarray data set.

Module ID	Number of Genes	*Z*-Summary Score	Top Functional Enrichment Terms (FDR *p*-value < 0.05)	Promoter Motif Enrichment (FDR *p*-value < 0.05)	Cell Type Marker Enrichment (FDR *p*-value < 0.05)
Random	1000	-0.450372394	-	-	-
1	262	0.951510976	Sarcomere, muscle structure development	-	-
2	209	1.967152607	Extracellular matrix organisation	EKLF(Zf)	-
3	137	0.864232356	Sequence specific DNA binding, AP2 transcription factors	-	-
4	54	0.097738923	-	-	-
5	133	0.342079228	Homeobox genes	-	-
6	212	-0.201216548	L27 protein domain	-	-
7	98	1.004032939	-	-	-
8	67	0.62219677	Vacuolar membrane	-	-
9	1493	3.032384176	-	-	-
10	57	-0.720174553	-	-	-
11	319	-0.828726101	Mismatch repair	-	-
12	163	0.101290609	-	-	-
13	668	3.95602259	G-protein coupled receptor signalling pathway	-	Neuron
14	81	0.842726312	Gland morphogenesis, Notch	Hoxb4(Homeobox)	-
15	82	1.741071825	Transcription factor binding	-	-
16	62	5.804071733	Ribosome, nonsense mediated decay	GFX, ZBTB33(Zf), ERG(ETS)	-
17	59	1.92925347	Synapse part, regulation of synaptic plasticity	-	-
18	189	-0.849139273	PI3K/AKT activation	GRE(NR)	-
19	1221	0.552830789	Cholesterol biosynthesis	-	-
20	381	5.071793968	Immune response	SpiB(ETS), ELF3(ETS), PU.1(ETS), IRF1(IRF), IRF8(IRF), PU.1-IRF(ETS:IRF), EWS:ERG-fusion(ETS), ELF5(ETS), IRF3(IRF), IRF2(IRF), ISRE(IRF), EHF(ETS), PU.1:IRF8(ETS:IRF), EBF(EBF), SPDEF(ETS)	Microglia
21	97	-0.689019253	Semaphorin receptor activity, integral component of membrane	-	-
22	127	0.53275753	-	-	-
23	309	1.081006115	Zinc Finger C2H2	GATA3(Zf)	-
24	103	0.322883371	Oxidative phosphorylation, fatty acid metabolism	-	-
25	55	-0.250719822	-	-	-
26	90	2.432423753	Regulation of MAPK cascade	-	-
27	69	-0.222869961	Oxidative phosphorylation	-	-
28	59	0.023104171	Pattern specification process, cardiovascular system development	-	-
29	171	0.553682784	Phototransduction	-	-
30	549	-0.624680799	Response to DNA damage	ETS1	-

The *Z*-Summary preservation score is a statistic that aggregates various *Z*-statistics obtained from permutation tests of the coexpression network to test whether network properties such as density and connectivity in the zebrafish co-expression network are preserved in an independent co-expression network constructed from human brain gene expression data. In this analysis, 200 permutations were used. *Z*-summary scores less than 2 indicate no preservation, while scores between 2 and 10 indicate weak-to-moderate evidence of preservation. The top functional enrichment and cell type marker enrichment terms are used to give insight into possible biological functions represented within each module. Cell type marker enrichment gene sets are from MSigDB, while functional enrichment terms are from Gene Ontology and MSigDB gene sets. The “Random” module is a random sample of 1,000 genes in the zebrafish co-expression network expected to show non-significant preservation (*Z*-summary < 2) in the human co-expression network. Shaded rows indicate zebrafish gene modules identified as showing significant preservation in the human network.

### Several pathological changes in the heterozygous mutant zebrafish brains are similar to those in human AD brains

**Fig 7:**
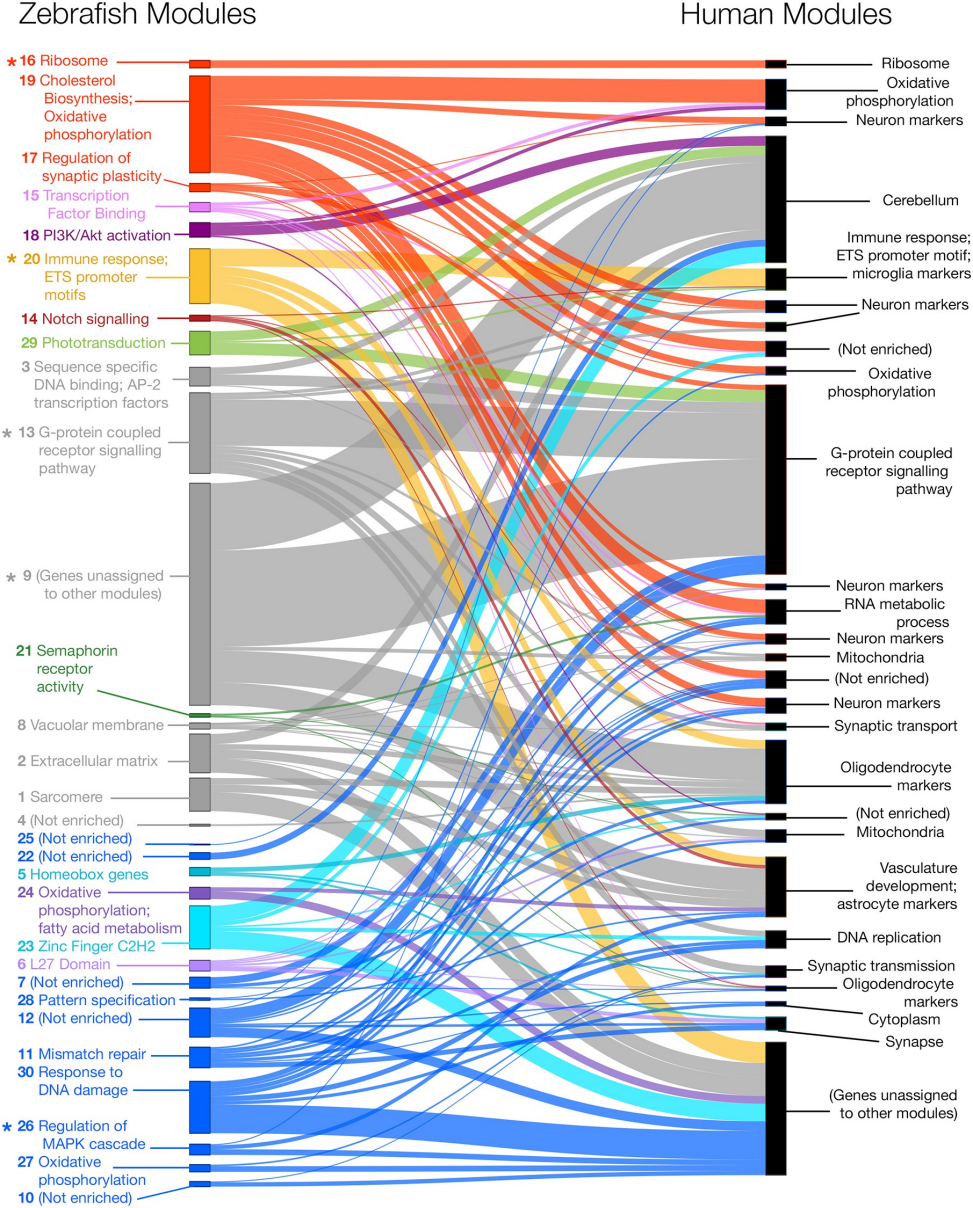
Module overlap between co-expression networks constructed using zebrafish and human brain gene expression data. Zebrafish and human co-expression networks were constructed using 7,118 genes that were orthologs in zebrafish and humans and expressed in brain gene expression data. Modules of co-expressed genes were separately identified for both the zebrafish and human co-expression networks, resulting in 30 modules in the zebrafish network (left) and 27 modules in the human network (right). Several zebrafish modules (indicated with asterisks) were found to have *Z*-summary preservation score > 2, indicating statistically significant weak-to-moderate preservation of these modules (i.e. genes in these modules still tend to be co-expressed) in the human brain co-expression network. Four out of five of these modules also showed statistically significant functional enrichment. See Table 1 for more details on the *Z*-summary preservation scores and functional enrichment for each module in the zebrafish co-expression network.

There are several methods for assessing whether modules are preserved across two independent gene co-expression networks constructed using the same genes [49]. The most easily interpretable method is to compare directly the assignment of equivalent genes to modules identified in each network. The resulting overlap in gene co-expression patterns across the two networks can be visualised using a Sankey diagram (Fig 7). Overall, the gene co-expression patterns in the zebrafish brain appear to be broadly similar to the gene co-expression patterns in the human brain, despite differences in RNA-seq platform and brain regions used, which would be expected to make the networks less comparable. A more sophisticated method of assessing module preservation involves using permutation-based *Z*-statistics to test whether certain properties of modules (e.g. density, connectivity) defined in one co-expression network are preserved in another network [49]. *Z*-statistics for each module property can be summarised into a *Z*-summary score, with *Z*-summary scores less than 2 indicating no module preservation, scores between 2 and 10 indicating weak to moderate module preservation, and scores above 10 indicating strong preservation [49]. When comparing zebrafish and human brain co-expression networks, four of the 30 zebrafish modules (**16**, **20**, **13**, **26**) have *Z*-summary scores between 2 and 10, indicating weak to moderate preservation in the human co-expression network (Table 1, S9 Table). While modules **16** (enriched in functions relating to ribosome and nonsense mediated decay) and **13** (enriched in G-protein coupled receptor activity) do not show significant differences between heterozygous mutant and wild-type brains as they age, modules **20** and **26** display distinct coordinated changes in expression during aging of wild-type brains. Module **20** genes are enriched in immune response functional terms and tend to be up-regulated with aging (correlation *p*-value 0.01), while module **26** genes are enriched in terms relating to regulation of the MAPK cascade and tend to be down-regulated with aging (correlation *p*-value 0.05, Fig 6). Importantly, these coordinated gene expression changes appear to be lost in aged heterozygous mutant brains (correlation *p*-values of 0.3 and 0.3 respectively), suggesting the K97fs mutation in *psen1* may contribute to alterations in at least these biological functions. Notably, module **26** which is enriched in immune response functions also displays significant enrichment in ETS and IRF promoter motifs (see Table 1, all FDR-adjusted *p*-values < 0.05). The equivalent module in the human co-expression network also displays enrichment in these particular motifs, suggesting that the regulation of immune and microglial gene expression responses is likely well conserved between aged zebrafish and human brains.

### Aged heterozygous mutant brains possess increased abundance of microglia

**Fig 8:**
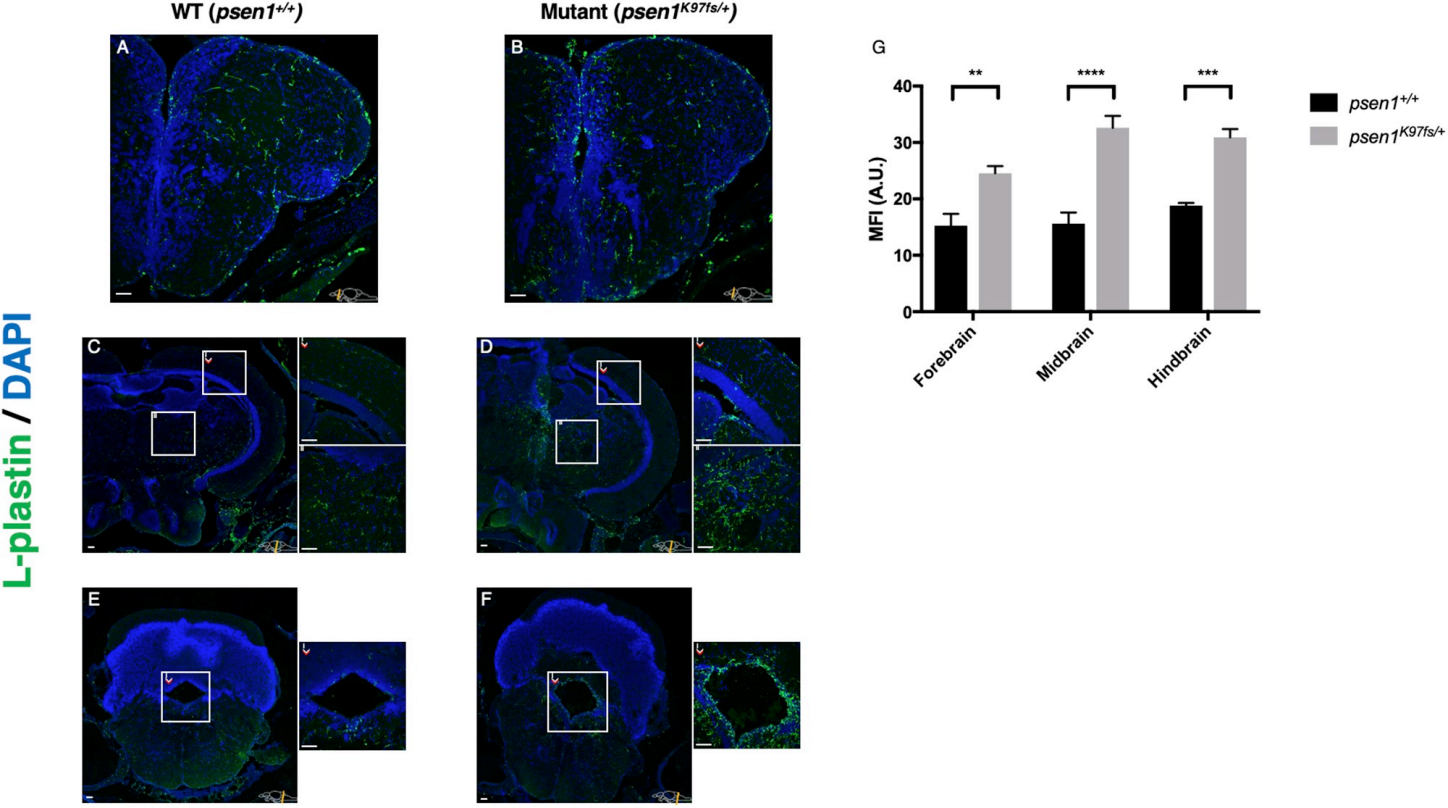
Cells expressing L-plastin are more abundant across the heterozygous mutant (*psen1*^*K97fs/+*^) zebrafish brain than in wild type siblings at 24 months. Immunostaining for the pan-leukocyte marker L-plastin supports increased numbers of microglia in the forebrain (**A-B**), midbrain (**C-D**) and hindbrain (**E-F**). Increased microglial abundance is evident in *psen1*^K97fs/+^ heterozygotes in both ventricular (**D.i, F.i**) and parenchymal (**D.ii**) regions compared to wild types (**C**, **E**). **(G)** Significant differences in MFI were observed between the forebrain, midbrain and hindbrain of psen1K97fs/+ and psen1+/+ fish; **p = 0.0048, ***p = 0.0005, ****p < 0.0001; two-way ANOVA with Sidak’s multiple comparisons test. Data presented as means with SEM. Scale bar 50 μm in all images.

The changes we observed in immune-microglia gene co-expression in the aged heterozygous mutant brains prompted us to ask whether differences might be observable in microglial form or even abundance. We used immunostaining for the pan-leukocyte marker L-plastin to detect microglia on sections of fixed brain material from 24-month-old wild type and heterozygous mutant zebrafish (Fig 8). An increased abundance of cells expressing L-plastin was evident in *psen1*^*K97fs/+*^ heterozygotes in both ventricular (Fig 8C.i and 8E.i) and parenchymal (Fig 8C.ii and 8F.i) regions compared to wild type brains (Fig 8D.i and 8D.ii). We observed significant differences in mean fluorescent intensity (MFI) of the image in the L-plastin channel indicating increased abundance of cells expressing L-plastin in the forebrain, midbrain and hindbrain regions of heterozygous mutant and wild type fish (Fig 8G, *p* = 0.0048, *p* = 0.0005, *p*<0.0001 respectively; two-way ANOVA with Sidak’s multiple comparisons test). This immunostaining was also capable of distinguishing between distinct morphologies of microglia in the ventricular (amoeboid “activated” morphology) and parenchymal (ramified morphology) regions in the zebrafish brain (**Fig H in** S1 File) although there was no obvious variation in morphology observed between heterozygous mutant and wild type brains.

### Molecular changes in the aged heterozygous mutant zebrafish brains occur without obvious histopathology

Teleosts (bony fish) such as the zebrafish show impressive regenerative ability following tissue damage that includes repair of nervous tissue. Previous attempts to model neurodegenerative diseases in adult zebrafish have failed to show cellular phenotypes [50]. Also, zebrafish are thought unlikely to produce the Aβ peptide [51] that many regard as central to AD pathological mechanisms [52]. The analyses described in this paper support that a fAD mutation mimicking PS2V formation may accelerate aspects of brain aging and promote a shift in aged heterozygous mutant brains towards an altered, pathological state of gene and protein expression. We therefore made histopathological comparisons of aged (24 months) wild type and heterozygous mutant brains equivalent to those used in our ‘omics analyses. Analysis of various brain regions using markers of aging, senescence and amyloid accumulation (lipofuscin, senescence-associated β-galactosidase, and Congo Red staining respectively) revealed no discernible differences (see Materials and methods and **Figs I-K in** S1 File). This is consistent with the lack of neurodegenerative histopathology observed in a heterozygous knock-in model of a *PSEN1* fAD mutation in mice [25].

## Discussion

**Fig 9:**
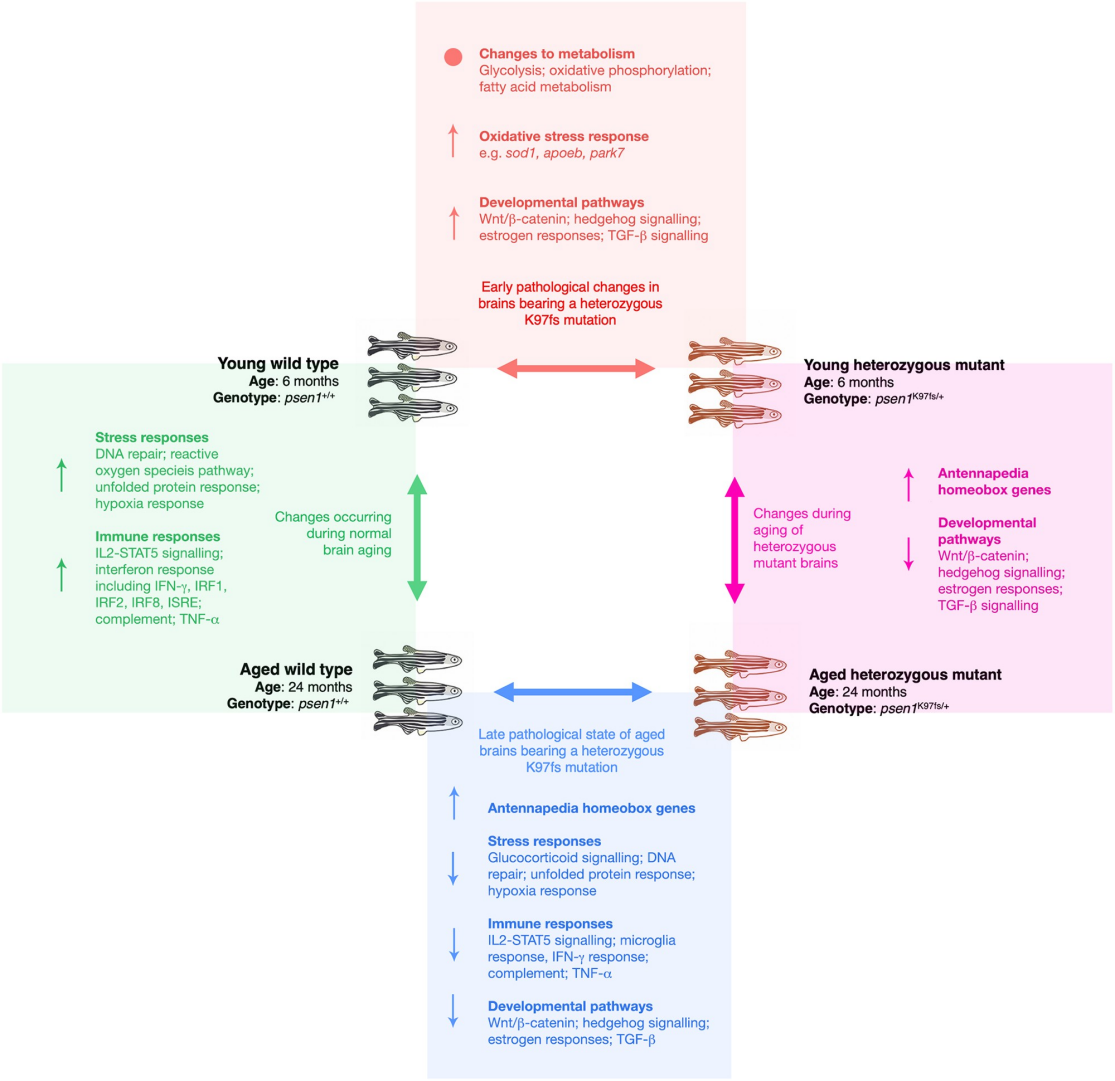
Summary of the molecular changes in the brains of zebrafish due to aging and/or the K115fs-like mutation (*psen1*^K97fs/+^). For each of the four pairwise comparisons shown, the summarised molecular changes (↑ = overall increased, ↓ = overall decreased, • = significant alterations but not in an overall direction) were inferred from a combination of the following analyses: functional enrichment analysis of differentially expressed genes and proteins, promoter motif enrichment analysis of differentially expressed genes, gene set enrichment analysis of differentially expressed genes, and weighted co-expression network analysis of the gene expression data.

Fig 9 summarises the main molecular changes that occur with aging and heterozygous mutation.

### Evidence of increased stress long preceding AD

We identified a subset of ‘inverted’ genes that are up-regulated in young heterozygous mutant brains, but down-regulated in aged heterozygous mutant brains. Although this pattern might be overlooked, similar patterns have been observed in human cases. Patients with Mild Cognitive Impairment, pre-clinical AD, or Down Syndrome (who often develop AD in adulthood) initially display increased expression of particular genes, which show decreased expression when AD symptoms become more severe [23, 53–55]. Collectively, results from these studies and our heterozygous mutant zebrafish suggest that early increases in brain activity likely precede AD symptoms in both *PSEN1*-mutation carriers and more general cases of AD. Evidently, to find strategies for preventing AD progression while patients are still asymptomatic, it is important to understand the causes of this increased gene activity in the brain.

Our results suggest that stress responses likely contribute to early increases in brain activity for fAD mutation carriers. In heterozygous mutant zebrafish, the inverted gene expression pattern seems to arise from altered glucocorticoid signalling. In humans, chronically increased glucocorticoid signalling in the brain can lead to glucocorticoid resistance, whereby the brain is unable to increase glucocorticoid signalling even during stressful conditions [56, 57]. We did not confirm whether glucocorticoid signalling and cortisol levels were altered in zebrafish brains *in vivo*. However, many of the inverted genes possess glucocorticoid receptor elements in their promoters, with one particular inverted gene (*fkbp5)* encoding a protein which is known to bind directly to the glucocorticoid receptor to negatively regulate its activity. Previous studies in humans demonstrate that *fkbp5* levels are highly responsive to chronic stress and stress-related diseases (e.g. bipolar disorder; depression in AD [58]), implying that *fkbp5* expression is a sensitive marker of glucocorticoid signalling. Our analysis supports this idea, with *fkbp5* mRNAs showing a significant difference in expression between heterozygous mutant and wild type brains (logFC = 2.1, FDR*p* = 1.77e-06 in young heterozygous mutant vs wild type; logFC = -3.9, FDR*p =* 3.16e-08 in aged heterozygous mutant vs wild type). Aside from altered glucocorticoid signalling, we also found altered gene expression patterns associated with diverse biological changes in heterozygous mutant zebrafish brains. If we assume that these heterozygous mutant zebrafish model some aspects of human AD, then these alterations may offer insight into early changes in the brains of human fAD-mutation carriers and, potentially, other individuals predisposed to AD. The brains of young heterozygous mutant zebrafish exhibit changes relating to developmental signalling pathways (Wnt/β-catenin signalling, hedgehog signalling, TGF-β signalling), stress and immune responses (DNA repair, IL2-STAT5 signalling, complement system, IFN-γ response, inflammatory response), hormonal changes (early and late estrogen responses, androgen response), and energy metabolism (glycolysis, oxidative phosphorylation). Appropriate regulation of these biological processes is critical for brain function, so it is unsurprising that disruption of these processes in the brain has been linked previously to various pathological states, including early stages of neurodegeneration [59–62].

Quantifying protein abundance in young heterozygous mutant zebrafish brains revealed additional sources of early-life stress. In young heterozygous mutant zebrafish brains, proteins associated with oxidative stress responses and energy metabolism in mitochondria already demonstrated altered abundance. Overall, stress responses were increased, consistent with the RNA-seq data, and decreased abundance of metabolic and antioxidant proteins imply mitochondrial function was likely already impaired. Both increased oxidative stress and altered energy metabolism are known to be early events in AD [2, 63–69], consistent with the idea that these events may contribute to early stress responses in the brain.

### Involvement of microglia-mediated immune responses in AD

Our analysis identified two modules with altered gene co-expression patterns in both the aged heterozygous mutant zebrafish brains and post-mortem human AD brains. These modules demonstrated significant functional enrichment in immune and microglial responses (module **20** in the zebrafish network) and regulation of the MAPK cascade (module **26** in the zebrafish network), consistent with their well-established dysfunction in human AD [9, 36, 70, 71]. Gene co-expression changes associated with the immune-microglia responses and the MAPK cascade were evident in aged but not young heterozygous mutant brains, suggesting that these changes are likely to occur in later stages of AD pathogenesis. Our results are consistent with two independent studies involving co-expression analysis of AD brains by Miller et al. [47] and Zhang et al. [48] which also identified a prominent immune-microglia module demonstrating similar changes in gene co-expression in AD patients, despite differences in patient cohorts used, brain regions and tissue types sampled, RNA-seq or microarray platforms, and methodology used to construct the gene co-expression networks. Collectively, the results from these studies and our analysis support the involvement of microglia-mediated immune responses in late stages of AD pathogenesis.

Our analysis reveals additional insights that help explain the involvement of the immune-microglia module in AD. Promoter enrichment analysis of genes in the immune-microglia module indicates statistically significant enrichment in several known motifs. Interestingly, all of these motifs are binding sites for transcription factors from either the ETS (SpiB, ELF3, ELF5, PU.1, EHF) or IRF (IRF3, IRF8, IRF1) families. This finding is important, because 1) ETS and IRF transcription factor motifs are also enriched in the promoters of genes that are up-regulated with brain aging in wild type zebrafish, but not in genes that are up-regulated with brain aging in heterozygous mutant zebrafish. This suggests that the genes they regulate are important during normal brain aging and that their dysregulation may contribute to pathology. 2) ETS and IRF transcription factors are known to mediate critical biological functions, with ETS factors regulating cellular differentiation, proliferation, cell-cycle control, apoptosis, migration and mesenchymal-epithelial interactions [72, 73], and IRF factors mediating immune and other stress responses. Our results are consistent with those in a previous study by Gjoneska et al. [74] that analysed RNA-seq and ChIP-seq (chromatin immunoprecipitation sequencing) data from mouse and human brain tissues, which found that immune response genes were up-regulated in both the CK-p25 mouse model and in human sporadic AD, that these genes were enriched in ChIP-seq peaks corresponding to ETS and IRF transcription factor motifs, and that microglia-specific activation was likely responsible for these gene expression changes.

Immunohistochemistry on sections from aged brains to identify L-plastin-expressing cells, (thought to represent microglia), revealed an increased abundance of these cells in heterozygous mutant fish compared to wild type fish but no obvious genotype-dependent differences in cell morphologies. The concentration of these cells in ventricle-proximal regions suggests an involvement with neural cell proliferation [75] which might occur in the regenerative zebrafish brain if the rate of cell turnover was increased due to pathological processes and this deserves future investigation. The increased abundance of L-plastin-expressing cells was not reflected in a noticeable increase in the mean expression of multiple microglial marker genes from the RNA-seq data. However, the RNA-seq data was derived from entire zebrafish brains and this may have obscured region-specific differences in microglial abundance, morphology, and activation.

Heterozygous mutant zebrafish in our study overall appear to recapitulate partially certain transcriptional and molecular changes that occur in more general cases of sporadic AD. Although revealing valuable insights, our comparison of the gene co-expression patterns in the zebrafish and human datasets is limited by inherent differences in species-level gene expression, differences in the brain regions and tissues sampled in each dataset, and difference in the RNA-seq platforms used to collect data, which has been previously shown to affect network properties including connectivity and density of modules [42]. In addition, the heterogeneity of sporadic AD would likely result in variation in gene expression patterns which may also confound our ability to identify reliably gene modules showing similar expression patterns across all samples. All of these differences would likely have contributed to decreasing our ability to detect preservation of modules between the zebrafish and human co-expression networks.

### AD-like gene expression changes can occur without amyloid pathology typically associated with AD

Somewhat surprisingly, the gene and protein expression changes observed in our aged heterozygous mutant zebrafish were not reflected in an obvious histopathology. However, this is consistent with an attempt to model neuronal ceroid lipofuscinosis in adult zebrafish [50] and with observations from heterozygous fAD mutation knock-in models in mice [25–27] (although, in general, mouse single heterozygous mutation brain histology phenotypes have not been reported). It is important to realise that differences in scale between the mass of a human brain and the brains of mice and zebrafish, (~1,000-fold and ~200,000 fold respectively) mean that any metabolic or other stresses in the small brains of the genetic models are likely exacerbated in the huge human brain [76]. Human brains also lack the regenerative ability of zebrafish, while mice and zebrafish both show sequence divergences in the Aβ regions of their APP orthologous genes greater than seen in most mammals [33, 77, 78]. Nevertheless, the heterozygous fAD-like mutation models of mice and (with this paper) zebrafish are probably the closest one can come to modelling AD in these organisms without subjectively imposing an opinion of what AD is by addition of further mutations or transgenes.

It is important to remember that the pathological role in AD of Aβ, neuritic plaques, and neurofibrillary tangles is still debated and that around one quarter of people clinically diagnosed with AD lack typical amyloid pathology upon post-mortem examination [79]. By the current definition, these people do not have AD [80] although this restrictive definition has been questioned [81, 82]. Many people also have brains containing high levels of Aβ [83] or Braak stage III to VI neurodegeneration [79] without obvious dementia. Thus the connection between amyloid pathology, histopathological neurodegeneration and Alzheimer’s disease dementia is unclear. Our data indicate that the AD cellular pathologies may occur subsequent to cryptic but dramatic changes in the brain’s molecular state (gene and protein expression) that are the underlying drivers of AD.

Finally, it is also important to acknowledge that the specific fAD mutation modelled in this study (K115fs of *PSEN2*) is an uncommon fAD mutation which produces novel alternative transcripts and splice isoforms, and it is unclear how pathogenic effects of this mutation might compare to other more common fAD mutations [84]. When beginning this research, we initially hypothesised that the alternative protein product PS2V (exon 6 deletion) produced from mutant K115fs *PSEN2* played a pathogenic role in AD. PS2V has previously been detected in sAD brains [29], and our previous research suggested dominant-negative effects of the functionally similar PS2V-like protein product produced from zebrafish *psen1* [32, 85]. Recent research suggests that some aberrant transcripts derived from the human K115fs mutant allele may, in fact, follow the "fAD mutation reading frame preservation rule" that is obeyed by all other fAD mutations in the *PSEN* genes [84]. If this is true, then our zebrafish model of K115fs is best regarded as illuminating the contribution that PS2V-mimicry by the K115fs mutation can make to its overall fAD phenotype. Nevertheless, our results indicate that the contribution made by such PS2V-mimicry is likely to be very significant. Our laboratory has been developing additional heterozygous mutant zebrafish modelling other forms of fAD mutation [86], and future analysis incorporating these zebrafish to produce a consensus co-expression network should help to identify and refine a “signature” of the transcriptome and proteome changes that cause fAD.

## Materials and methods

### Zebrafish husbandry and animal ethics

This study was approved under permits S-2014-108 and S-2017-073 issued by the Animal Ethics Committee of the University of Adelaide. Tübingen strain zebrafish were maintained in a recirculated water system.

### Generation of TALEN coding sequences and single stranded oligonucleotide

TALEN coding sequences were designed by, and purchased from, Zgenebio (Taipai City, Taiwan). The DNA binding sites for the TALEN pair targeting *psen1* were (5’ to 3’): left site, CAAATCTGTCAGCTTCT and right site, CCTCACAGCTGCTGTC (**Fig A in** S1 File). The coding sequences of the TALENs were provided in the pZGB2 vector for mRNA *in-vitro* synthesis. The single stranded oligonucleotide (ssoligo) sequence was designed such that the dinucleotide ‘GA’ deletion was in the centre of the sequence with 26 and 27 nucleotides of homology on either side of this site (**Fig A in** S1 File). The ssoligo was synthesized by Sigma-Aldrich (St. Louis, Missouri, USA) and HPLC purified. The oligo sequence was (5’ to 3’): CCATCAAATCTGTCAGCTTCTACACACAAGGACGGACAGCAGCTGTGAGGAGC (**Fig A in** S1 File).

### In-vitro mRNA synthesis

Each TALEN plasmid was linearized with *Not* I. Purified linearized DNA was used as a template for *in-vitro* mRNA synthesis using the mMESSAGE mMACHINE SP6 transcription kit (Thermo Fisher, Waltham, USA) as per the manufacturer’s instructions as previously described [85].

### Microinjection of zebrafish embryos

Embryos were collected from natural mating and, at the 1-cell stage, were microinjected with a ~3nl mixture of 250ng/μl of left and right TALEN mRNA and 200ng/μl of the ssoligo.

### Genomic DNA extraction of zebrafish tissue

#### Embryos

A selection of 10–20 embryos were collected at 24 hpf and placed in 150μl of a 50mM NaOH 1xTE solution and then incubated at 95°C until noticeably dissolved (10-20mins). The lysis solution was cooled to 4°C and 50μl of Tris solution (pH 8) was added. The mixture was then centrifuged at maximum speed for 2 mins to pellet cellular debris. The supernatant was transferred into a fresh microfuge tube ready for subsequent PCR.

#### Adult fin clips

For fin clips, adult fish were first anesthetised in a 0.16 mg/mL tricaine solution and a small section of the caudal fin was removed with a sharp blade. Fin clips were placed in 50μl of a 1.7 μg/ml Proteinase K 1xTE solution and then incubated at 55°C until noticeably dissolved (2-3hours). The lysis solution was then placed at 95°C for 5mins to inactivate the Proteinase K.

### Genomic DNA PCR and sequencing for mutation detection

To genotype by PCR amplification, 5 μl of the genomic DNA was used with the following primer pairs as relevant. Primers to detect wild type (WT) sequence at the mutation site: primer psen1WTF: (5’TCTGTCAGCTTCTACACACA*GA*AGG3’) (GA nucleotides in italics) with primer psen1WTR: (5’AGTAGGAGCAGTTTAGGGATGG3’). Primers to detect the presence of the GA dinucleotide deletion: primer psen1GAdelF: (5’AATCTGTCAGCTTCTACACACAAGG3’) with primer psen1WTR. To confirm the presence of the GA dinucleotide deletion mutation by sequencing of extracted genomic DNA, PCR primers were designed to amplify a 488 bp region around the GA mutation site: primer psen1GAsiteF: (5’GGCACACAAGCAGCACCG3’) with primer psen1GAsiteR: (5’TCCTTTCCTGTCATTCAGACCTGCGA3’). This amplified fragment was purified and sequenced using the primer psen1seqF: (5’ AGCCGTAATGAGGTGGAGC 3’). All primers were synthesized by Sigma-Aldrich. PCRs were performed using GoTaq polymerase (Promega, Madison, USA) for 30 cycles with an annealing temperature of 65°C (for the mutation-detecting PCR) or 61°C (for the WT sequence-detecting PCR) for 30 s, an extension temperature of 72°C for 30 s and a denaturation temperature of 95°C for 30 s. PCR products were assessed on 1% TAE agarose gels run at 90V for 30 mins and subsequently visualized under UV light.

### Whole brain removal from adult zebrafish

Adult fish were euthanized by sudden immersion in an ice water slurry for at least ~30 seconds before decapitation and removal of the entire brain for immediate RNA or protein extraction. All fish brains were removed during late morning/noon to minimise any influence of circadian rhythms.

### RNA extraction from whole brain

Total RNA was isolated from heterozygous mutant and WT siblings using the *mir*Vana miRNA isolation kit (Thermo Fisher). RNA isolation was performed according to the manufacturer’s protocol. First a brain was lysed in a denaturing lysis solution. The lysate was then extracted once with acid-phenol:chloroform leaving a semi-pure RNA sample. The sample was then purified further over a glass-fiber filter to yield total RNA. This procedure was formulated specifically for miRNA retention to avoid the loss of small RNAs. Total RNA was then sent to the ACRF Cancer Genomics Facility (Adelaide, Australia) to assess RNA quality and for subsequent RNA sequencing on the Illumina NextSeq platform as paired-end 100bp reads.

#### RNA extraction and cDNA synthesis from entire brains for digital PCR

Total RNA was extracted using the QIAGEN RNeasy Mini Kit according to the manufacturer’s protocol. The RNA was DNase-treated using RQ1 DNase (Promega) according to the manufacturer’s protocol prior to cDNA synthesis. Equal concentrations of total RNA from each brain were used to synthesise first-strand cDNA by reverse transcription with random priming (Superscript III kit; Invitrogen). cDNA was RNaseH treated before use in 3D Quant Studio Digital PCR.

#### Allele-specific digital quantitative PCR

Digital PCR was performed on a QuantStudio™ 3D Digital PCR System (Life Technologies, Carlsbad, California, USA). 20μL reaction mixes were prepared containing 9 μL 1X QuantStudio^™^3D digital PCR Master Mix (Life Technologies), 2 μL of 20X Sybr^®^ dye in TE buffer, 25ng cDNA per total reaction (determined from the RNA concentration under the assumption that single strand cDNA synthesis from total RNA was complete), 200nM of specific primers and 6.3 μL of nuclease-free water (Qiagen). 14.5μL of the reaction mixture was loaded onto a QuantStudio^™^3D digital PCR 20 K chip (Life Technologies) using an automatic chip loader (Life Technologies) according to manufacturer’s instructions. Loaded chips underwent thermo-cycling on the Gene Amp 9700 PCR system under the following conditions: 96°C for 10 min; 45 cycles of 60°C for 2 min and 98°C for 30 sec; followed by a final extension step at 60°C for 2 min. After thermo-cycling, the chips were imaged on a QuantStudio^™^ 3D instrument [87, 88]. Primers used for *psen1* allele detection were: wild-type allele forward 5’ CTACACACAGAAGGACGGACAGC 3’, K97fs allele forward 5’ TCTGTCAGCTTCTACACACAAGGA 3’ and both were paired with a common reverse primer 5’ GCCAGGCTTGAATCACCTTGTA 3’.

#### PCR test for aberrant splicing in the region of the K97fs mutation in zebrafish psen1

Total RNA was extracted from each 24-month-old zebrafish brain using the QIAGEN RNeasy mini Kit (QIAGEN, Hilden, Germany). 250ng of total RNA from each brain was then used to synthesise 20μL of first-strand cDNA by reverse transcription (SuperScript III kit, Invitrogen, Camarillo, California, USA). 10ng of each cDNA preparation (a quantity calculated from the RNA concentration on the assumption that reverse transcription of RNA into cDNA was complete) was used to perform PCR using Phusion high-fidelity DNA polymerase (New England Biolabs, Ipswich, Massachusetts). Each 25μL PCR reaction contained 0.2mM of deoxyribonucleotide triphosphates (dNTPs), 0.4μM of each PCR primer, 1 unit of Phusion polymerase and 10ng of zebrafish brain cDNA template. PCR cycling was performed with 35 cycles of a denaturation temperature of 95°C for 30s, then an annealing temperature of 60°C for 30s and then an extension temperature of 72°C for 2 minutes. PCR products were electrophoresed through a 1% agarose gel in 1×TAE buffer for separation and identification.

### Protein extraction and proteomic analysis of adult brain

#### Sample preparation

Freshly removed entire adult zebrafish brains were lysed under denaturing conditions in 7 M urea (Merck, Darmstadt, Germany) plus complete protease inhibitors (Roche) using a Bioruptor (Diagenode, Seraing, Belgium) in ice cold water. Samples were quantified using the EZQ protein assay (Life Technologies) and the extracts were trypsin-digested using the FASP method [89]. Protein samples were then sent to the Adelaide Proteomics Centre (Adelaide, Australia) for quantification and data acquisition.

#### Data acquisition

Nano-LC-ESI-MS/MS was performed using an Ultimate 3000 RSLC system (Thermo Fisher Scientific) coupled to an Impact HD^™^ QTOF mass spectrometer (Bruker Daltonics, Bremen, Germany) via an Advance Captive Spray source (Bruker Daltonics). Peptide samples were pre-concentrated onto a C18 trapping column (THC164535, Thermo Fisher) at a flow rate of 5 μL/min in 2% (v/v) ACN 0.1% (v/v) FA for 10 minutes. Peptide separation was performed using a 75μm ID 50 cm C18 column (THC164540, Thermo Fisher) at a flow rate of 0.2 μL/minute using a linear gradient from 5 to 45% B (A: 5% (v/v) ACN 0.1% (v/v) FA, B: 80% (v/v) ACN 0.1% (v/v) FA) over 180 minutes. MS scans were acquired in the mass range of 300 to 2,200 m/z in a data-dependent fashion using Bruker’s Shotgun Instant Expertise^™^ method (singly charged precursor ions excluded from acquisition, CID from 23% to 65% as determined by the m/z of the precursor ion).

#### Data analysis

The acquired peptide spectra were identified and quantified using the mass spectrometry software MaxQuant with the Andromeda search engine against all entries in the non-redundant UniProt database (protein and peptide false discovery rate set to 1%). The MaxQuant software allows for the accurate and robust proteomewide quantification of label-free mass spectrometry data [90].

### RNA-seq analysis

#### Data processing

We used *FastQC* [91] to evaluate the quality of the raw paired-end reads. Using *AdapterRemoval* [92], we trimmed reads and removed adapter sequences. From the *FastQC* reports, some over-represented sequences in the raw and trimmed reads corresponded to ribosomal RNA, possibly from insufficient depletion during library preparation. We removed ribosomal RNA sequences *in silico* by aligning all trimmed reads to known zebrafish ribosomal RNA sequences and discarding all reads that aligned. Next, we used *HISAT2* [93] to align reads to the Ensembl zebrafish genome assembly (GRCz10). Using *Picard* [94] and the MarkDuplicates function, we removed optical and PCR duplicates from the aligned reads. To quantify gene expression, we used *FeatureCounts* [95], resulting in a matrix of gene expression counts for 32,266 genes for the 12 RNA-seq libraries.

#### Differential gene expression analysis

Differential gene analysis was performed in R [96] using the packages *edgeR* [97] and *limma* [98–100]. We retained 18,296 genes with >1.5 cpm in at least 6 of the 12 RNA-seq libraries. We then calculated TMM-normalisation factors to account for differences in library sizes and applied the RUVs method from the *RUVseq* package [101] to account for a batch effect with one factor of unwanted variation (*k* = 1). Differential gene expression analysis was performed using *limma*. We considered genes differentially expressed if the FDR-adjusted *p*-value associated with their moderated *t*-test was below 0.05. We used the *pheatmap* R package [102] to produce all heatmaps.

#### Gene set testing

We downloaded the Hallmark gene set collection from MSigDB v6.1 [38]. Using *biomaRt* [103, 104], we converted human Entrezgene identifiers to zebrafish Entrezgene identifiers. To perform gene set testing, we applied the fast rotation gene set testing (FRY) method [105] for each comparison. We considered all gene sets with non-directional (Mixed) FDR < 0.05 as differentially expressed. To obtain estimates of the proportions of up-regulated and down-regulated genes for each significant gene set, we used the ROAST [106] method with 9,999 rotations with the ‘set.statistic’ option set to ‘mean’, to maintain consistency with the results obtained from FRY.

#### Promoter motif analysis

We performed promoter motif enrichment analysis using *HOMER* [107, 108] and downloaded a set of 364 zebrafish promoter motifs from published ChIP-seq experiments, as collated by *HOMER* authors, using the command ‘configureHomer.pl -install zebrafish-p’. We retained default parameters with the findMotifs.pl program with the following modifications: the 18,296 Ensembl genes considered as expressed in the differential gene expression analysis were specified as the background; and promoter regions were defined as 1500 bp upstream and 200 bp downstream of the transcription start site. We defined motifs as being significantly enriched in a set of genes if the Bonferroni-adjusted *p*-value was less than 0.05.

### LC-MS/MS analysis

#### Data processing

Raw MS/MS spectra were analysed using *MaxQuant* (V. 1.5.3.17). A False Discovery Rate (FDR) of 0.01 for peptides and a minimum peptide length of 7 amino acids was specified. MS/MS spectra were searched against the zebrafish UniProt database. *MaxQuant* output files for the 6-month-old and 24-month-old samples were processed in separate batches with the *MSStats* R package [109] due to an unresolvable batch effect. Briefly, peptide intensities were log_2_-transformed and quantile normalised, followed by using an accelerated failure time model to impute censored peptides. Peptide-level intensities were summarised to protein-level intensities using Tukey’s median polish method. This resulted in 2,814 peptides (summarised to 534 proteins) for the 6-month-old data and 3,378 peptides (summarised to 582 proteins) for the 24-month-old data. After summarisation, both sets of protein intensities were combined, quantile normalised and filtered to retain the 323 proteins that were detected across all samples.

#### Differential protein analysis

Differential protein abundance analysis was performed using *limma* [110] using moderated *t*-tests. Proteins were identified as being differentially abundant if FDR-adjusted p-values were below 0.05. Over-representation analysis using the ‘goana’ and ‘kegga’ functions from *limma* were used to test for enriched gene ontology terms and KEGG pathways respectively.

### Gene co-expression network analysis

#### Network construction

We used the *WGCNA* R package to construct co-expression networks for our zebrafish RNA-seq data and a processed human RNA-seq dataset from the Mayo RNAseq study [41]. The human RNA-seq data consists of 101 bp paired-end reads sequenced with the Illumina HiSeq 2000 platform and derived from cerebellum and temporal cortex samples from North American Caucasian subjects with either AD (n = 86), progressive supranuclear palsy (PSP, n = 84), pathological aging (PA, n = 28) or controls lacking neurodegeneration (n = 80). The Mayo RNAseq study authors performed read alignment and counting using the *SNAPR* software with the GRCh38 reference human genome and Ensembl v77 gene models, and provided TMM-normalised gene counts as output by the *edgeR* package [97, 111]. We matched zebrafish genes to human homologous genes via orthologous Ensembl gene identifiers and retained genes that were expressed in both the human and zebrafish datasets, leaving 8,396 genes for network construction. To reduce noise during network construction, we calculated connectivities for each gene in each dataset and retained 7,576 genes with connectivities above the 10th percentile of all connectivities. To construct approximately scale-free weighted networks, the Pearson correlation was calculated between each pair of genes, and the resulting correlation matrix was raised to the soft-thresholding power of 14 to produce a signed adjacency matrix for each dataset [43]. Next, we applied a transformation to obtain a measure of topological overlap for each pair of genes. Lastly, we hierarchically clustered genes in each dataset based on the measure 1—Topological Overlap. To identify modules of co-expressed genes, we used the Hybrid Tree Cut method from the *dynamicTreeCut* package [112] with default parameters except for the following modifications: minimum module size set at 40 genes, 0.90 as the maximum distance to assign previously unassigned genes to modules during PAM (Partioning Around Medoids) stage, and the deepSplit parameter to 1 for both the human and zebrafish datasets.

#### Network analysis

We assessed functional enrichment of each module using default settings in the *anRichment* R package. We assessed promoter motif enrichment using HOMER as described earlier. To calculate the correlation between modules and phenotypic traits, we calculated the hybrid-robust correlation between the first principal component of each module and four binary variables defining the experimental conditions [113]. We evaluated the preservation of zebrafish modules in the human network and vice versa using the modulePreservation function from *WGCNA*, which uses a permutation-based approach to determine whether module properties (e.g. density, connectivity) are preserved in another network [49]. We also used the Sankey diagram functionality in the *networkD3* package to visualise overlap between zebrafish and human modules [114].

#### Network visualisation

To visualise networks, we imported edges and nodes into *Gephi* and applied the OpenOrd algorithm with default settings [115]. We coloured the nodes (genes) based on their assigned modules from *WGCNA*.

### *psen1*^K97fs/+^ vs. wild type L-plastin immunostain

#### Anti-L-plastin immunostain

Frozen cryosections of adult *psen1*^K97fs/+^ (n = 3 fish) or *psen1*^+/+^ (n = 4 fish) brains were dried for >1hr at room temperature, then rehydrated in 1x PBS for >30 min. Sections were then washed twice with 0.3% Triton X-100 in 1x PBS (PBS-Tx 0.3%) for 15 min at room temperature (RT). Sections were subsequently incubated with polyclonal rabbit anti-L-plastin primary antibody [116, 117] (a kind gift from Prof. Dr. Michael Brand, Centre for Regenerative Therapies, Technische Universität Dresden) at 1:2500 concentration in PBS-Tx 0.3%, overnight at 4°C in a humid chamber. Sections were then washed 3 x 20 min in PBS-Tx 0.3% at RT, followed by 1 h incubation at RT with goat anti-rabbit Alexa 488 secondary antibody (Thermo Fisher, 1:750 in PBS-Tx 0.3%) alongside DAPI at 1:5000 concentration. Sections were subsequently washed once for 10 min with PBS-Tx 0.3%, then twice for 20 min with 1x PBS at RT, then mounted with 50% glycerol in 1 x PBS.

#### Imaging

Z-stacks of brain sections were acquired on a Leica TCS SP8 confocal microscope equipped with a HyD detector, and using the Leica LASX software suite. Stacks were captured at 1024 x 1024 resolution at scanning speed of 600, with bidirectional X scanning active. No averaging or accumulation was applied to stack acquisitions, and laser power and gain were kept constant throughout image acquisition. Overview stacks were captured with either a 10x dry objective (midbrain, hindbrain overviews) or a 20x oil-immersion objective (forebrain overviews), while higher magnification stacks were acquired with a 40x water-immersion objective. ~3 stacks were captured per fish (one of each forebrain, midbrain and hindbrain at approximately equal levels).

#### Image processing and statistical analysis

Stacks were opened in Fiji 2 (https://imagej.net/Fiji/Downloads), and split into individual channels. The green channel (corresponding to L-plastin in all stacks) was max-projected and the MFI of the entire image was recorded using the Measure function. Statistical analysis was conducted in Prism 7 (GraphPad); MFI between brain regions in *psen1*^K97fs/+^ and *psen1*^+/+^ fish was compared via two-way ANOVA with Sidak’s multiple comparisons test. Statistical significance was defined as *p*<0.05, with all data presented as means with SEMs.

### Histological analysis

#### Tissue preparation

Two-year-old adult zebrafish heterozygous for the psen1K97fs mutation and their wild type siblings were sacrificed by immersion in ice-water, then tails were nicked to exsanguinate the fish and prevent blood clotting on neural tissue. The dorsal neurocranium was subsequently resected to expose the brain. Fish were then decapitated and heads were incubated in a decalcification solution (100 ml 0.5M EDTA, 22 g sucrose, 11 ml 10x phosphate buffered saline solution, PBS) for four hours on a slow shaker at room temperature. Decalcified heads were then fixed overnight in 4% paraformaldehyde in phosphate buffer (PFA in PB), at 4°C on a slow shaker. Heads were then embedded in a sucrose-gelatin medium (20% sucrose, 8% cold-water fish gelatin in 1x PBS), frozen on dry ice and cryosectioned at 16 μm thickness on a Leica CM3050-S cryostat. Serial sections were mounted on SuperFrost Plus microscope slides (Menzel-Gläser). Sections were subsequently dried at room temperature for four hours, and then stored at -20°C until staining. Prior to all stains, sections were retrieved from -20°C and brought to RT, then rehydrated in 1x PBS.

#### Senescence-associated β-galactosidase (saβgal) staining

Sections were prefixed with 4% PFA in PB for one hour in a humid chamber at room temperature, then washed twice for 15 minutes with 0.3% Triton X-100 in 1x PBS (PBS-Tx (0.3%)). The pH of sections was then equilibrated with two 30 minute washes with 1x PBS at pH 5.5 (all washes were performed in a humid chamber). Sections were then stained for 16 hours at 37°C in a humid chamber in staining solution (2 mM MgCl2, 5 mM K3Fe(CN)6, 5 mM K3Fe(CN)6·3H2O, 1 mg/ml X-gal, with the remaining volume made up of 1x PBS at pH 5.5). Following incubation, staining was arrested by a 20-minute wash in 4% PFA in PB at room temperature. Sections were then washed well in PBS at pH 5.5 and mounted in 50% glycerol. Sections were then imaged on an Olympus Provis AX70 widefield microscope with an Olympus DP70 camera, with images acquired at 4080x3072 resolution.

#### Congo Red staining for amyloid

Following rehydration in 1x PBS, sections were stained for 20 minutes in Congo Red staining solution (0.5% Congo Red in 50% ethanol). Sections were then rinsed in distilled water, and quickly differentiated by dipping five times in an alkaline alcohol solution (1% NaOH in 50% ethanol). Sections were rinsed for 1 minute in distilled water, then mounted in 50% glycerol. Sections were imaged in both brightfield and birefringence on a Leica Abrio polarising microscope at 1024x1024 resolution.

#### Autofluorescent detection of lipofuscin

Following rehydration in 1x PBS, sections were mounted in 50% glycerol and imaged confocally on a Leica TCS SP8 invert confocal laser scanning microscope with a Leica HyD hybrid detector using 20x and 63x oil-immersion objectives. Lipofuscin has an emission maximum at 590 nm when excited at 488 nm; samples were thus excited with a 488nm laser at power 5.00, and the detector was gated for emission wavelengths between 560–700 nm. Images were acquired at 1024x1024 resolution with gain at 137.8%.

## Supporting information

S1 File Supporting figures and text. (PDF)

S1 Table Differential gene expression results for each comparison. (XLSX)

S2 Table Differential protein abundance results for each comparison. (XLSX)

S3 Table Summary of functional enrichment results for groups of genes identified in differential gene expression analysis. (PDF)

S4 Table Full gene ontology analysis results for groups of genes identified in differential gene expression analysis. (XLSX)

S5 Table Summary of promoter motif enrichment analysis in groups of genes identified from differential gene expression analysis. (PDF)

S6 Table Full enrichment results of known zebrafish promoter motifs in groups of genes identified from differential gene expression analysis. (XLSX)

S7 Table List of zebrafish genes containing the GRE (glucocorticoid receptor element) motif. These genes have a 90% match for the GRE motif in the region 1500bp upstream to 200bp downstream of the transcription start site.
(XLSX)

S8 Table Differentially expressed Hallmark MSigDB gene sets for each comparison identified using the FRY (fast rotation) method. Proportions of up- and downregulated genes in each set were estimated using ROAST (which FRY approximates). Differentially expressed gene sets were defined as having “Mixed FDR” < 0.05.
(XLSX)

S9 Table Zebrafish module functional enrichment results using anRichment R package. (XLSX)

S10 Table Zebrafish module promoter motif enrichment results using HOMER software. (XLSX)

S11 Table Human module functional enrichment results using anRichment R package. (XLSX)

S12 Table Promoter motif enrichment analysis of the immune-microglia enriched module in the zebrafish network. (XLSX)

S13 Table *Z*-score calculation results for preservation of zebrafish co-expression network properties in human co-expression network. (XLSX)
