# Supplementary material for: Accelerated brain aging towards transcriptional inversion in a zebrafish model of the K115fs mutation of human PSEN2
Source: PLoS One. 2020 Jan 24;15(1):e0227258. doi: 10.1371/journal.pone.0227258 (PMC6980398; doi:10.1371/journal.pone.0227258)
Supplement: S1 File — (PDF) [file pone.0227258.s001.pdf]

S1 File. Supporting Figures and Text.

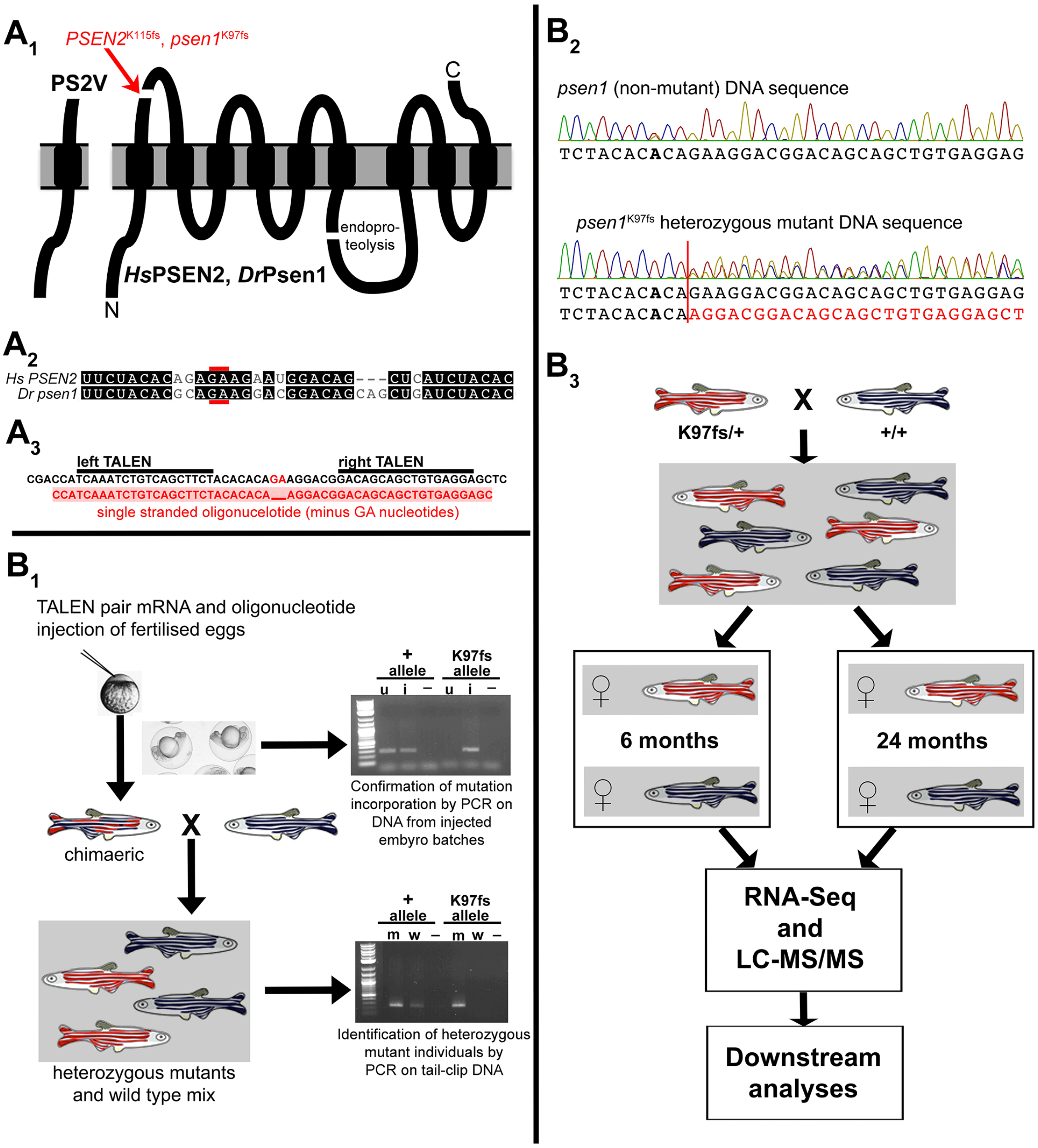

Fig A. Gene editing in zebrafish to produce the *psen1* K97fs mutation.

A1. PRESENILIN and PS2V protein structure. The lipid bilayer is shaded grey and the transmembrane domains are indicated by black ovals. The site of the Homo sapiens (Hs) *PSEN2* K115fs and Danio rerio (Dr) *psen1* K97fs mutation is indicated in red.

A2. A section of the Hs *PSEN2* and Dr *psen1* nucleotide alignment around the K115fs (K97fs) mutation site.

A3. Section of genomic Dr *psen1* sequence around the GA target site with TALEN binding sites indicated and the single stranded oligonucleotide sequence below.

B1. Flow diagram for creation of the Psen1 K97fs heterozygous mutation model. Upper agarose gel image is of amplified genomic DNA from 24hpf uninjected (u) embryos, injected (i)

embryos and water negative control (-). PCR reactions containing either wild type (wt) detecting or mutation (mut) detecting primers. Lower agarose gel image is of amplified genomic DNA from adult tailclips from a wild type (w), a K97fs heterozygous germline mutant (m) and water negative control (-). The amplified DNAs are from PCR reactions containing either wild type (wt) detecting or mutation (mut) detecting primers.

B_2. Chromatographs of a section of genomic *psen1* sequence around the K97fs deletion site for wild type DNA and for K97fs heterozygous mutant DNA. Red sequence is the altered DNA sequence after GA dinucleotide deletion.

B_3. A K97fs heterozygous mutant (red stripes) was pair mated with a wild type (Tübingen) to produce a tank of fish that is ~1:1 mutant : non-mutant. At 6 months and 24 months of age, brains were removed from female K97fs heterozygous mutant adults and non-mutant adult siblings. Mutant and non-mutant brains were subjected to either RNA-seq or LC-MS/MS procedures followed by downstream analysis of the data.

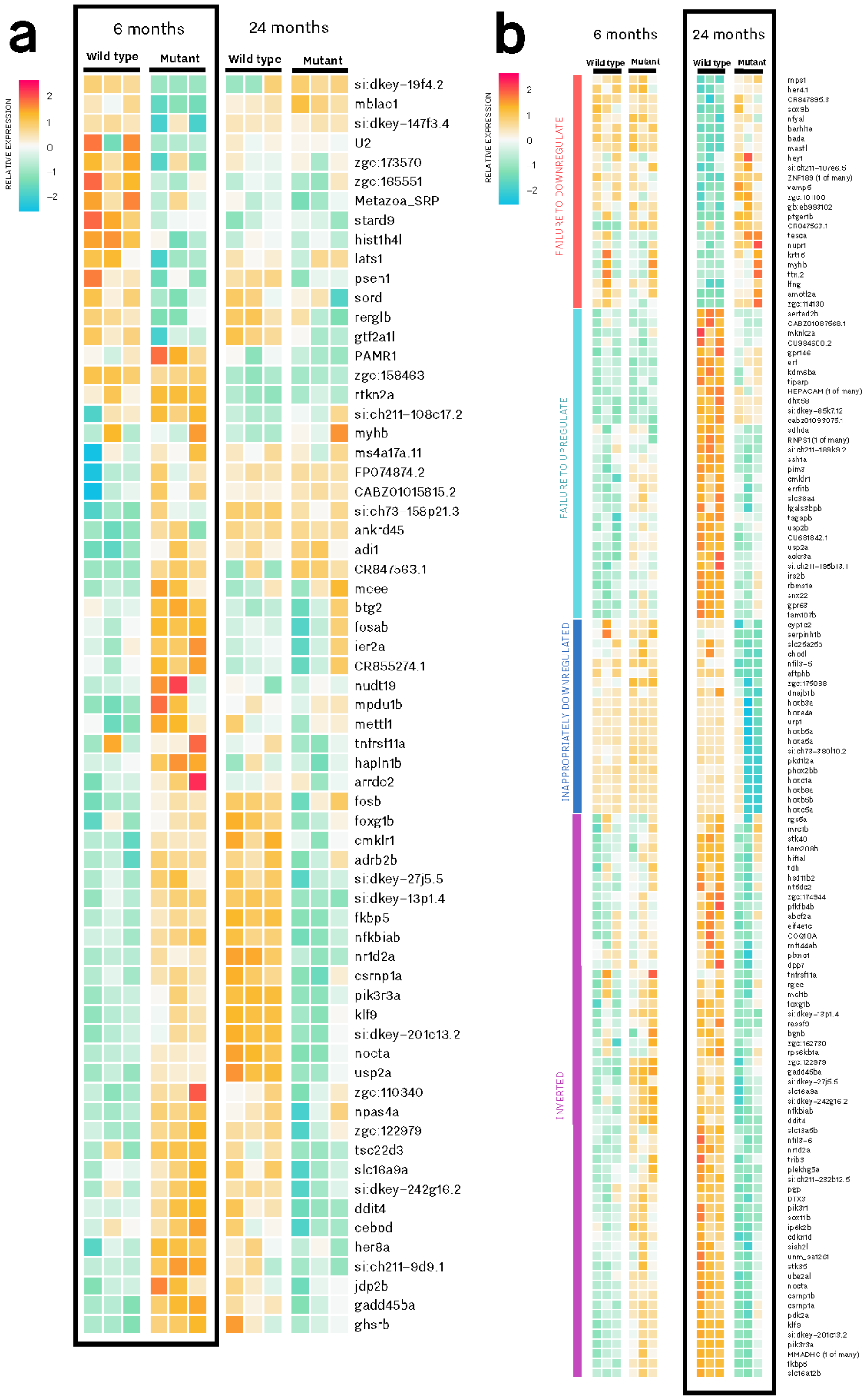

Fig B. Gene expression changes in the brains of heterozygous mutant (*psen1*$^{K97fs/+}$) zebrafish compared to wild type (*psen1*$^{+/+}$) siblings at 6 and 24 months of age. Genes shown in the heatmaps are restricted to those with False Discovery Rate [FDR]-adjusted p-value < 0.05 and absolute $\log_2$ fold change > 0.5 for the following comparisons: **a.** 6-month-old heterozygous mutant vs. 6-month-old wild type zebrafish brains. **b.** 24-month-old mutant vs. 24-month-old wild type zebrafish brains. Gene expression patterns for the opposite age group are also shown in both **a** and **b** (not in black boxes). Genes are hierarchically clustered with Euclidean distance measure. Biological interpretations are proposed for the clusters of genes in **b**.

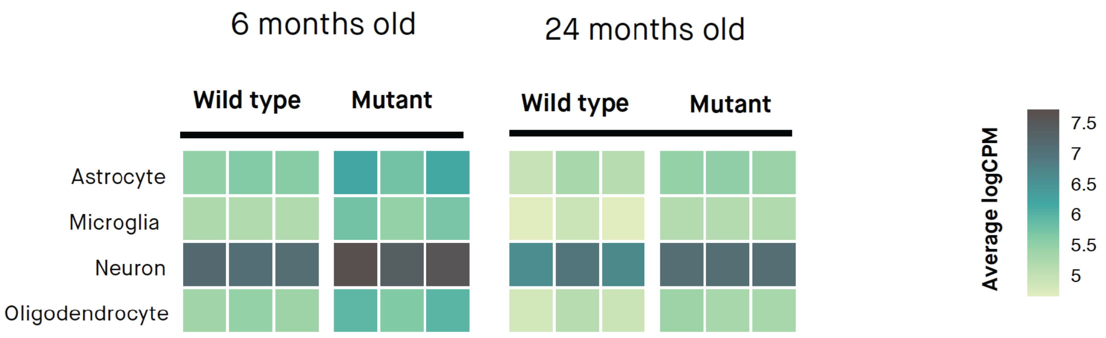

Fig C. Average expression (logCPM) of gene markers for various neural cells (astrocyte, microglia, neuron, oligodendrocyte) in whole brains of heterozygous mutant and wild type zebrafish. Representative marker genes for microglia were obtained from [1] while gene markers for the remaining neural cells were obtained from [2]. The number of genes used to calculate the average gene expression (in logCPM) was 41 (astrocyte), 99 (microglia), 77 (neuron) and 78 (oligodendrocyte). Although this method is limited in that it does not account for the significant diversity within these broader cell types nor regional brain differences, this level of analysis suggests that broadly, the average expression of gene markers for the major neural cell types does not appear to change much across experimental conditions. In addition, no obvious outlier samples were evident.

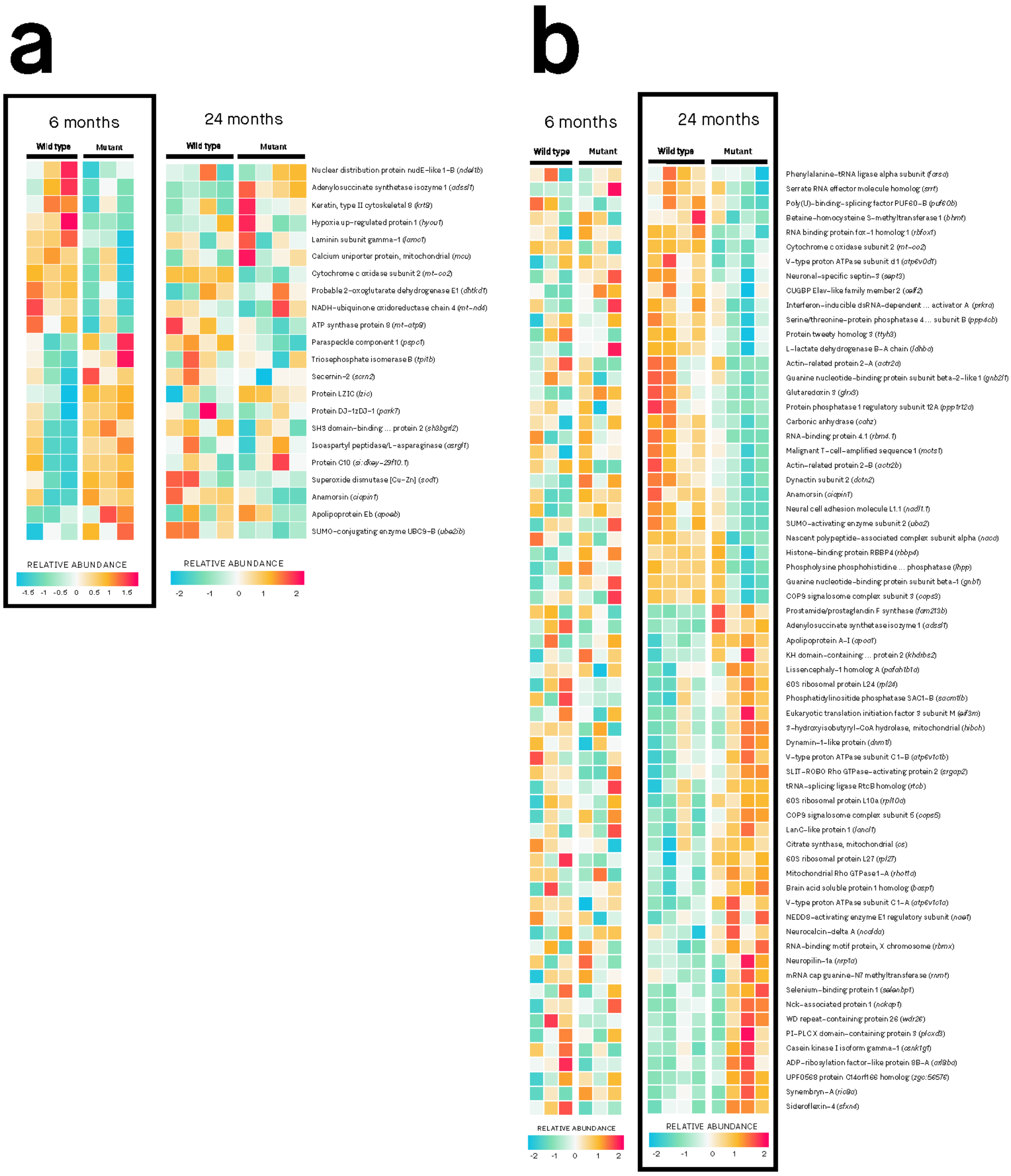

Fig D. Proteins distinguishing heterozygous mutant (*psen1*$^{K97fs/+}$) from wild type (*psen1*$^{+/+}$) siblings. a. Differentially abundant proteins at 6 months of age. **b.** Differentially abundant proteins at 24 months of age. Proteins were considered differentially abundant if the False Discovery Rate (FDR)-adjusted *p*-value of their moderated *t*-test was below 0.05. The protein abundance of the opposite age group is also shown for **a** and **b**. Colours of heatmap cells represent relative protein abundance levels, scaled across the samples in each age group.

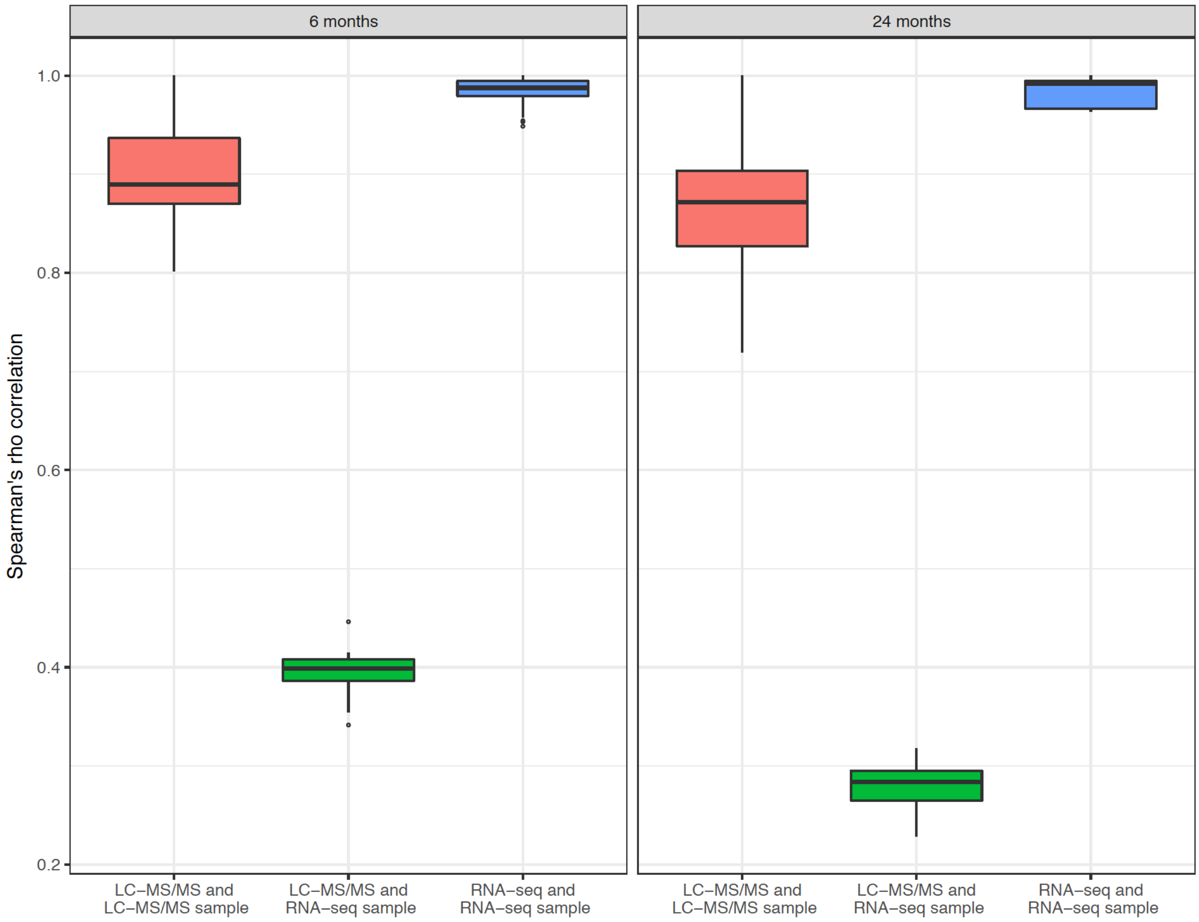

Fig E. Boxplots of Spearman's rho (rank) correlation between RNA-seq (gene expression) and LC-MS/MS (protein abundance) data for each sample. By using matching Ensembl gene identifiers to overlap the normalised and filtered LC-MS/MS and RNA-seq data sets, we identified 219 proteins that were reliably quantified in both data sets. Overall, the correlation between the abundance level of these proteins and their corresponding gene expression counts in the RNAseq data was weakly positive (Spearman's rho 0.34-0.45 for LC-MS/MS and RNA-seq data for 6- month-old zebrafish brains; Spearman's rho 0.24-0.31 for LC-MS/MS and RNA-seq data for 24- month-old zebrafish brains).

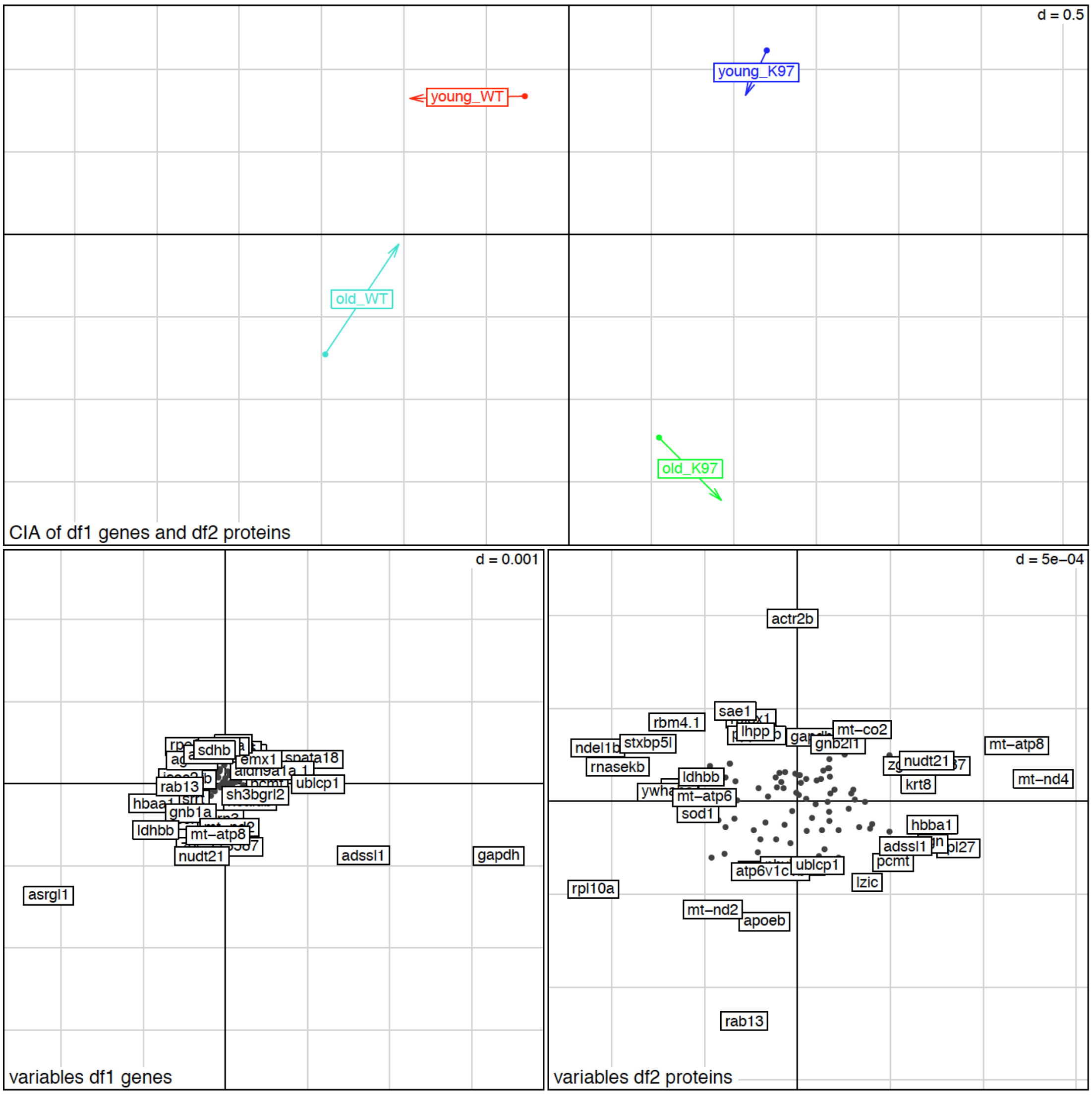

Fig F. Co-inertia analysis (CIA) of matched protein-gene pairs from the LC MS/ MS (protein abundance) and RNA-seq (gene expression) data sets. Co-inertia analysis is a multivariate statistical method that has been used to integrate transcriptomic and proteomic data to identify relationships between these datasets [3]. First, a multivariate analysis transformation (in this case, non-symmetric CA, NSC) was separately applied to the proteomic and transcriptomic data sets. Co-inertia analysis then uses the NSCs to identify common trends or relationships in the data sets by selecting axes that maximise the square covariance [3]. In the top panel, the base of the arrow represents the gene expression, the tip of the arrow represents the protein abundance, and the length of the arrow is proportional to the overall similarity between the gene and protein expression/abundance levels. The bottom two panels plot the matched genes (left) and proteins (right) used in the CIA.

Overall, the four biological conditions (young wild type, young mutant, aged wild type, aged mutant) possess sufficiently different gene expression and protein abundance patterns to separate them from other biological conditions. However, the lengths of the arrows suggest moderate differences between gene expression and protein abundance patterns for each biological condition. In CIA, the global correlation between data sets can be summarised by the RV coefficient which takes values from 0 (no correlation) to 1 (perfect correlation). The RV coefficient of the RNA-seq and LC-MS/MS data sets in this case is 0.428, indicating moderate global similarity for the proteins and genes that can be matched.

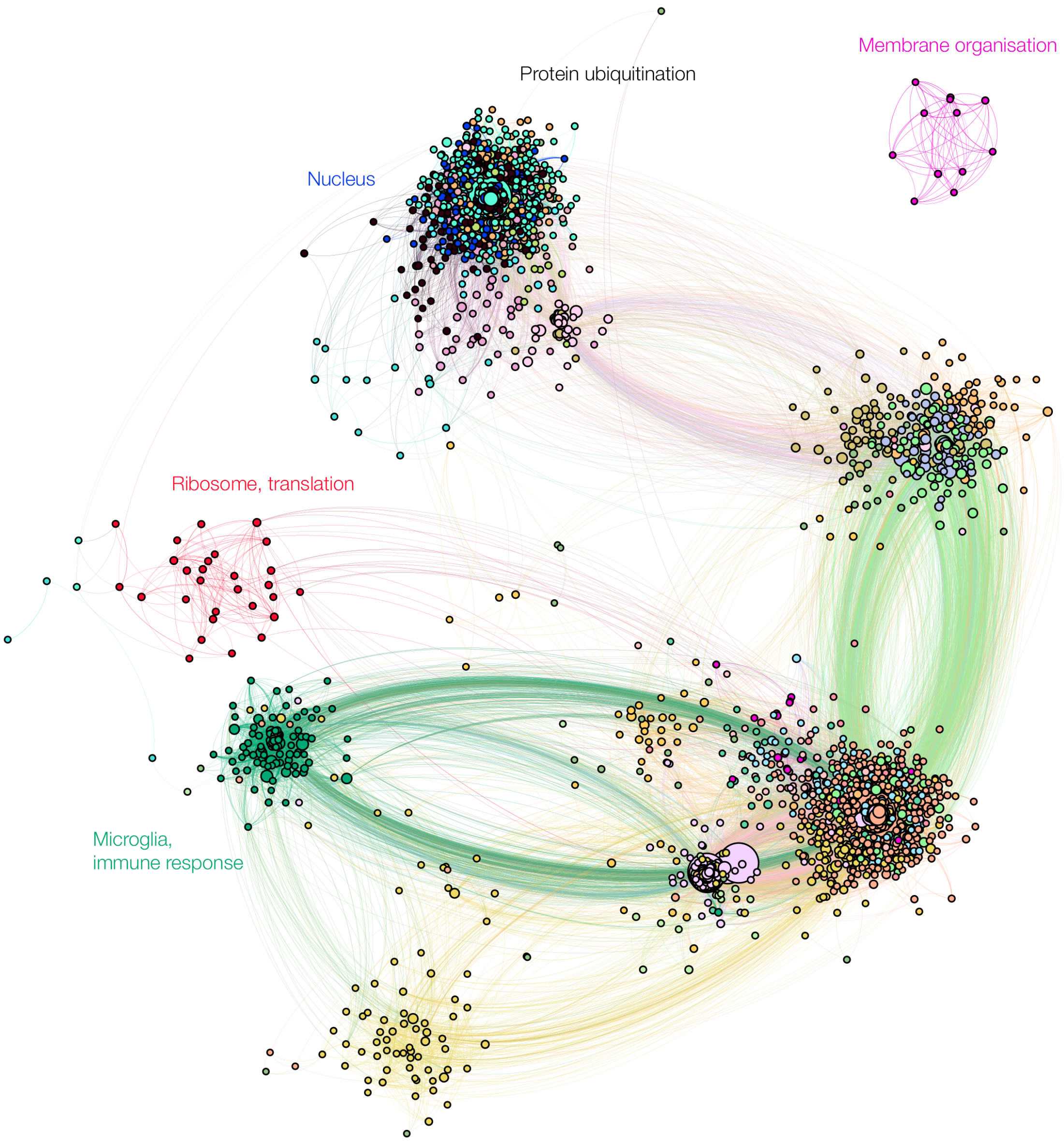

Fig G. Human brain gene co-expression network. Each node represents one gene, with node size proportional to the number of connected nodes (co-expressed genes). Edges represent co-expression between two genes, with edge weight proportional to the strength of co-expression. The co-expression network is a signed adjacency matrix constructed from RNA-seq data from cerebellum and temporal cortex brain tissue derived from patients from the Mayo RNAseq study who have been diagnosed with either Alzheimer's disease, progressive supranuclear palsy, pathological aging, or controls without neurodegenerative diseases. Only nodes with at least four connections are shown. Gene "modules" are groups of genes with similar expression patterns across the samples. In this network, 27 gene modules were identified using a hierarchical clustering and branch cutting method. Each module is coloured in a different colour. The five labelled modules were identified to have statistically significant preservation in the zebrafish brain gene co-expression network (*Z*-summary scores > 2). The labels represent the enriched functions of the genes within each module.

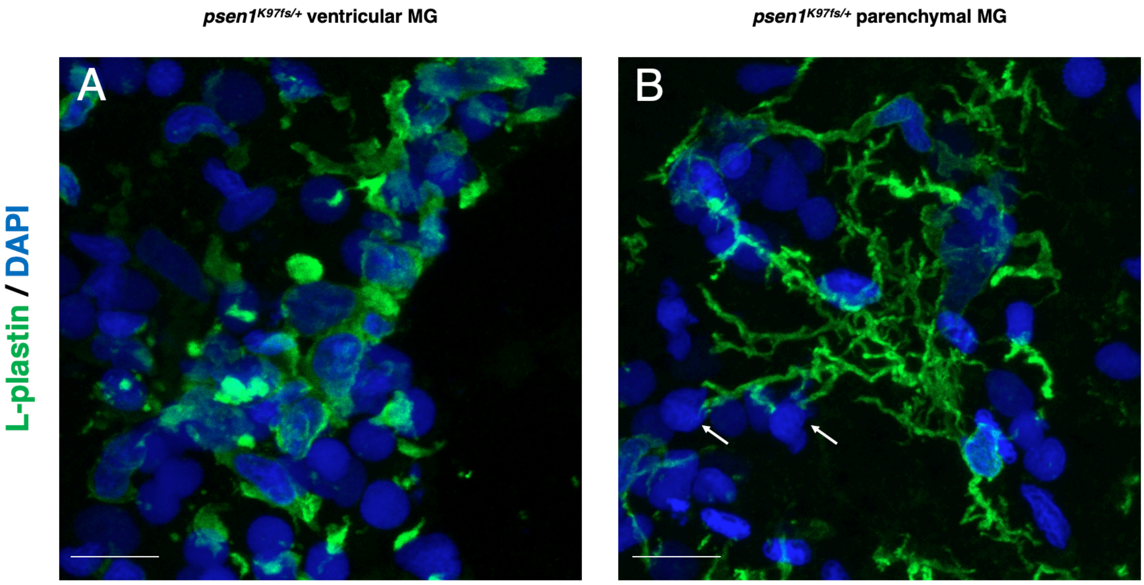

Fig H. Distinct morphologies of cells expressing L-plastin between ventricular and parenchymal regions in heterozygous mutant (*psen1*$^{K97fs/+}$) zebrafish brains at 24 months. **A.** Ventricular microglia broadly exhibit an amoeboid ‘activated’ morphology. **B.** Parenchymal microglia broadly exhibit a ramified morphology, with processes contacting neighbouring cells (arrows). Similar region-specific differences in morphology were observed in wild type brains Scale bar 10 µm.

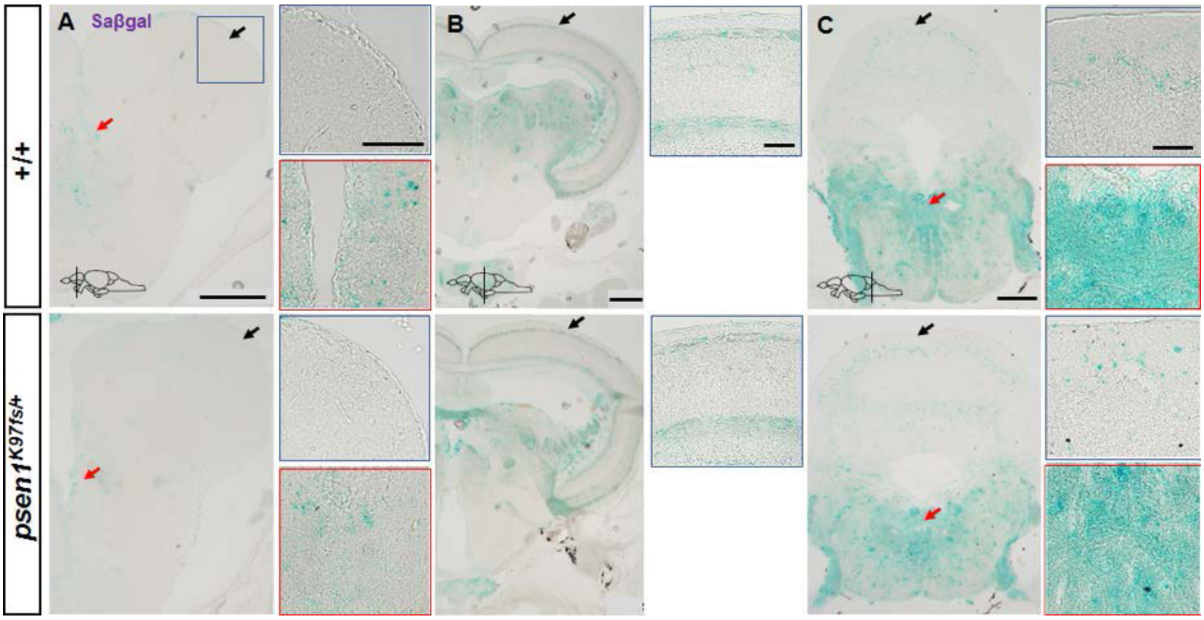

Fig I. saβgal staining in two-year-old heterozygous mutant (*psen1*$^{K97fs/+}$) and wild type (*psen1*$^{+/+}$) zebrafish brain. A. Cross section of telencephalon. Black arrows show very weak saβgal staining in the dorsal telencephalon. Red arrows show weak saβgal staining in neurons in nuclei lateral to the telencephalic ventricle. **B.** Cross section of the mesencephalon/diencephalon showing hypothalamus and the optic tectum. Black arrows show weak saβgal staining in superficial and deep layers of the optic tectum. Staining is primarily in layers containing fibers. **C.** Cross section of the cerebellum. Black arrows show weak saβgal staining in neurons in molecular and Purkinje cell layers. Red arrows show strong saβgal staining in neurons and fibres ventral to the rhombencephalic ventricle. Scale bar for overviews in A-C = 200 µm and 40 µm for the high magnification inset areas.

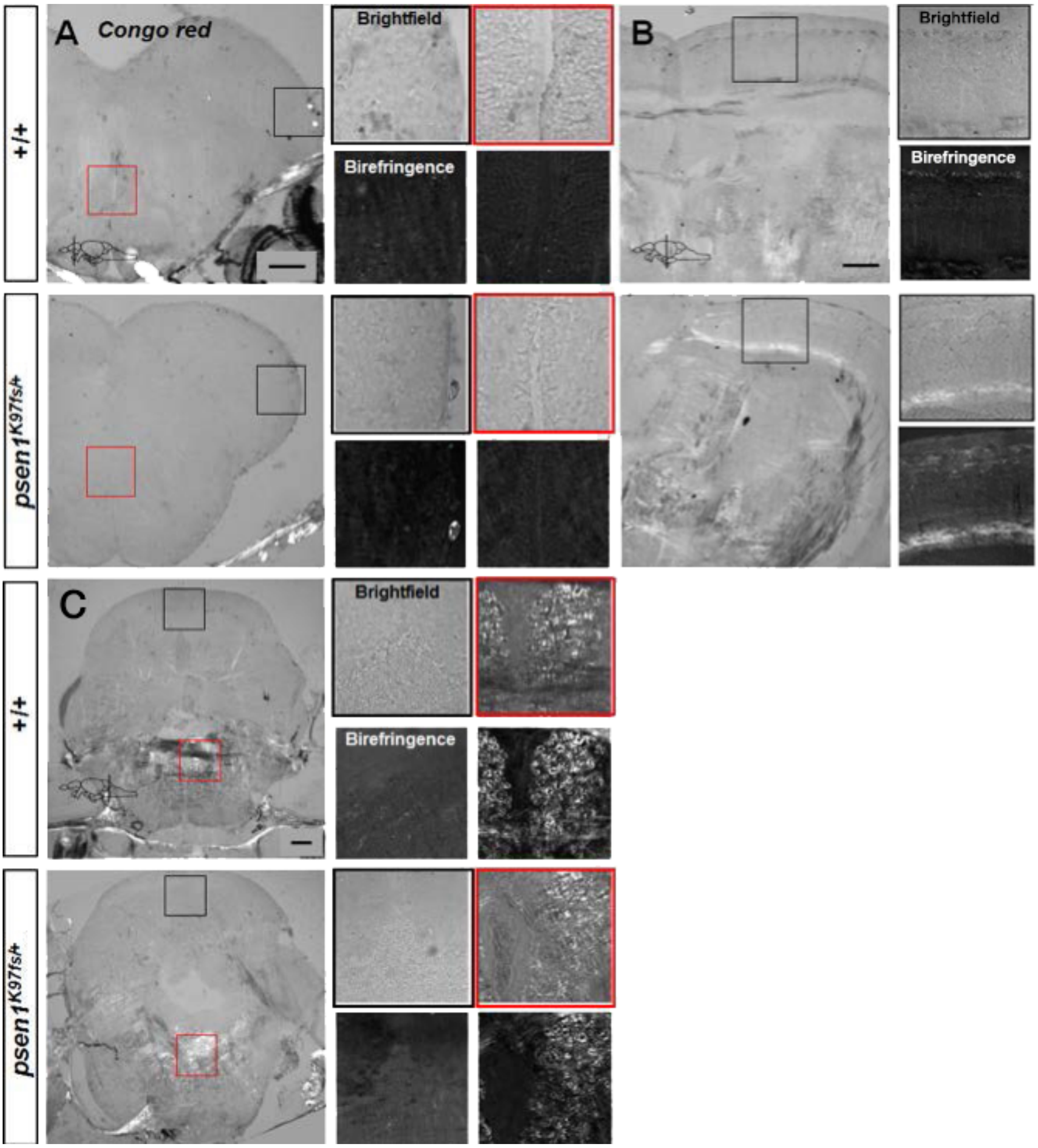

Fig J. Congo red staining in two-year-old heterozygous mutant (*psen1*$^{K97fs/+}$) and wild type (*psen1*$^{+/+}$) zebrafish brain. A. Cross section of telencephalon. Very weak or no Congo Red staining is observed in the dorsal telencephalon (bright field and birefringence). **B.** Cross section of the mesencephalon/diencephalon showing the optic tectum. Congo Red staining is seen as birefringence in a deep layer of the optic tectum (bright field and birefringence). Staining is primarily in layers containing fibers. **C.** Cross section of the cerebellum. Very weak or no Congo Red staining is seen in the cerebellum, while intense birefringence is observed in fibres of the median forebrain bundle ventral to the rhombencephalic ventricle. Scale bar for overviews in A-C = 100µm.

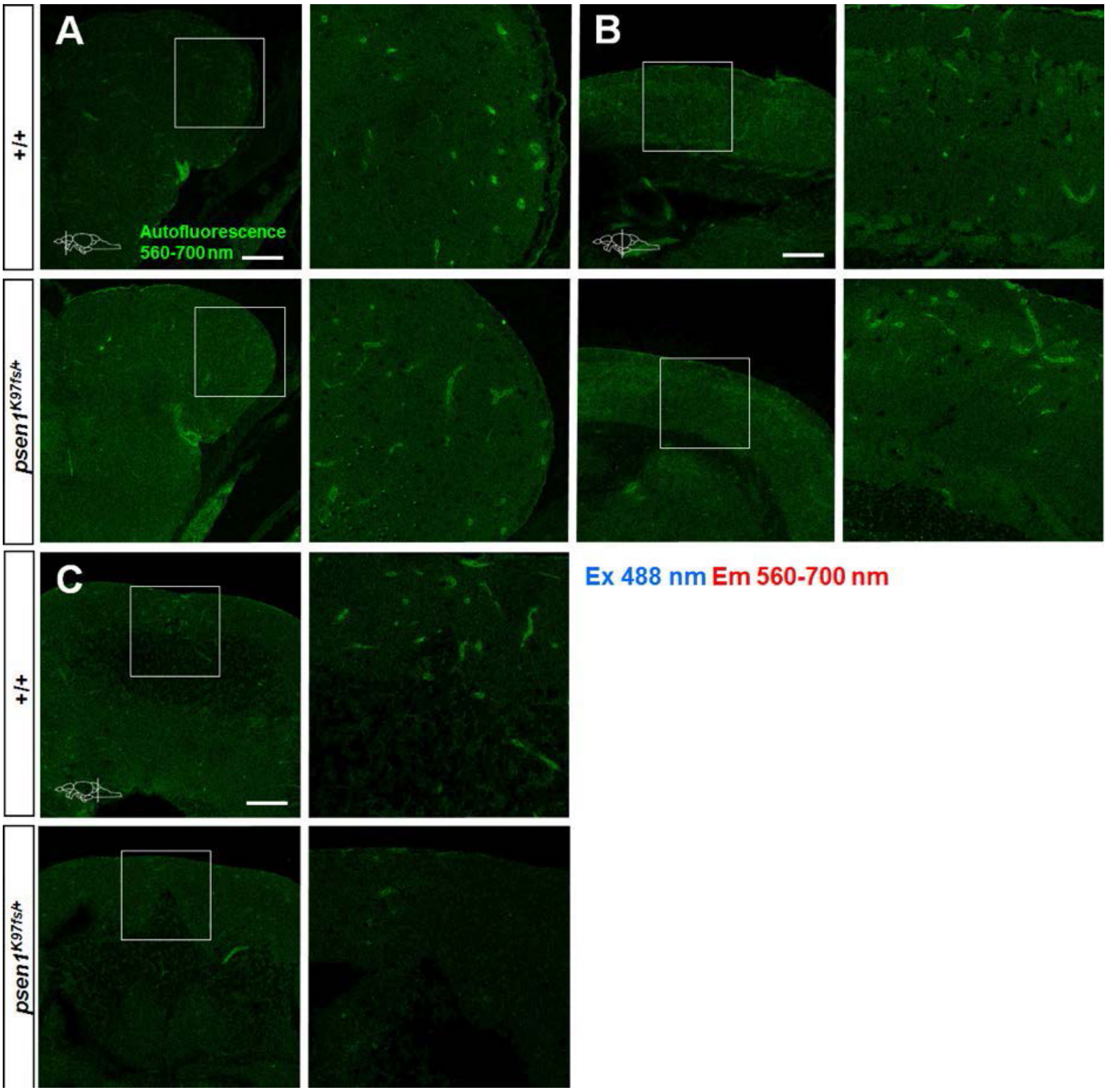

Fig K. Measurement of autofluorescence (Lipofuscin) in two-year-old heterozygous mutant (*psen1*$^{K97fs/+}$) and wild type (*psen1*$^{+/+}$) zebrafish brain. Excitation was performed using 488 nm laser light and autofluorescence was detected between 560 and 700 nm. **A.** Cross section of telencephalon. Weak autofluorescence was detected in the dorsal telencephalon. The boxed areas are shown at higher magnification to the right. **B.** Cross section of the mesencephalon/diencephalon showing the optic tectum. Weak autofluorescence is detected in all layers of the optic tectum**.** The boxed areas are shown at higher magnification to the right. **C.** Cross section of the cerebellum. Weak autofluorescence is detected in all layers of the cerebellum. The boxed areas are shown at higher magnification to the right. Scale bar in A-C = 100 µm.

References

1. Oosterhof N, Holtman IR, Kuil LE, van der Linde HC, Boddeke EW, Eggen BJ, et al. Identification of a conserved and acute neurodegeneration-specific microglial transcriptome in the zebrafish. Glia. 2017;65(1):138-49. Epub 2016/10/19. doi: 10.1002/glia.23083. PubMed PMID: 27757989; PubMed Central PMCID: PMCPMC5215681.

2. Lein ES, Hawrylycz MJ, Ao N, Ayres M, Bensinger A, Bernard A, et al. Genome-wide atlas of gene expression in the adult mouse brain. Nature. 2007;445(7124):168-76. Epub 2006/12/06. doi: 10.1038/nature05453. PubMed PMID: 17151600.

3. Fagan A, Culhane AC, Higgins DG. A multivariate analysis approach to the integration of proteomic and gene expression data. Proteomics. 2007;7:2162-71. doi: 10.1002/pmic.200600898.
