# Supplementary material for: Accelerated brain aging towards transcriptional inversion in a zebrafish model of the K115fs mutation of human PSEN2
Source: PLoS One. 2020 Jan 24;15(1):e0227258. doi: 10.1371/journal.pone.0227258 (PMC6980398; doi:10.1371/journal.pone.0227258)
Supplement: S3 Table — (PDF) [file pone.0227258.s004.pdf]

Supplementary Table 3. Summary of functional enrichment in different sets of genes identified from differential gene expression analysis of zebrafish brains. Up to the top three functional terms are included in this table; see Supplementary Table 3 for full analysis results. Functional terms are considered significantly enriched when the Bonferroni-adjusted *p*-value < 0.05. The 18,296 genes remaining after filtering in the differential gene expression analysis form the background gene set. Only genes with Entrezgene identifiers were included in the functional enrichment analysis.

Differentially expressed in 'aged mutant vs. aged wild type' (177 genes)

Enriched functional term	Description	Gene Names	% Genes with term	% Background genes with term	Bonferroni *p*-value
Homeobox Antennapedia CS (IPR001827, *Interpro domains*)	Antennapedia subfamily of homeobox genes	hoxb5b, hoxb3a, hoxb5a, hoxa5a, hoxc5a, hoxb8a, hoxa4a	6.19%	0.12%	1.18E-06
Homeobox CS (IPR017970, *Interpro domains*)	Conserved homeobox site	hoxb3a, hoxb5b, barhl1a, hoxc5a, hoxb5a, zgc:101100, hoxc1a, hoxa4a, hoxa5a, hoxb8a, phox2bb	9.73%	0.94%	0.000359
GSE18791 CTRL VS NEWCASTLE VIRUS DC 8H DN (*MSigDB*)	Genes down-regulated in comparison of control conventional dendritic cells (cDC) at 0 h versus cDCs infected with Newcastle disease virus (NDV) at 8 h.	amotl2a, gadd45ba, lgals3bpb, LOC566587, dhx58, phox2bb, mastl, csrnp1b, ddit4	12.86%	0.93%	0.000640

Differentially expressed in 'aged wild type vs. young wild type' (1,795 genes)

Enriched functional term	Description	Gene Names	% Genes with term	% Background genes with term	Bonferroni *p*-value
GSE19888 ADENOSINE A3R INH PRETREAT AND ACT BY A3R VS TCELL MEMBRANES ACT MAST CELL UP (*MSigDB*)	Genes up-regulated in HMC-1 (mast leukemia) cells: incubated with the peptide ALL1 and then treated with Cl-IB-MECA versus stimulation by T cell membranes.	psmb9a, tnfsf10, gbp2, ikzf1, hck, ifih1, irf7, psmb8a, psme1, si:ch1073-340j19.1, zgc:171731, nmi, mxe, si:ch211-197g15.8, irf9, si:ch211-160o17.2, zgc:92791, eif2ak2, tnfaip2a, lyn, mpeg1.1, mxc, tap1, dhx58, xaf1, si:ch211-219a4.3, samsn1b, lipg, b4galt5, gne, il2rga, tapbp.1, parp3, npc2, pik3ap1, rnf213a, gla, edem1	4.00%	0.88%	4.42E-12
GSE42021 CD24HI VS CD24INT TREG THYMUS DN (*MSigDB*)	Genes down-regulated in thymic T reg: CD24 high versus CD24 int.	abcb3l1, b2m, parp3, fosl2, tapbp.1, b2ml, si:dkey-201c1.2, mhc1uka, nfkbiab, sema3fa, tap1, gpr18, mxc, bcl2l13, tfg, calcoco2, rps6ka5, gbp1, erap1b, zgc:92791, mhc1zba, irf9, si:dkey-57h18.2, mxe, lgals3bpa, si:ch211-197g15.8, ctss2.2, hist2h2l, mhc1uba, itm2ba, psme1, erap1a, zgc:171731, nmi, ifit12, irf7, rbms1a, psmb8a, tnfsf10, psmb9a, ifih1	4.32%	1.27%	3.91E-08
Antigen processing and presentation	The process in which an antigen-presenting cell expresses antigen	b2m, tap1, cd74a, si:ch73-158p21.3, cd74b, mhc1uba, si:busm1-266f07.2, si:dkey-	1.38%	0.23%	7.81E-05

(GO:0019882, *GO Biological Process*)	(peptide or lipid) on its cell surface in association with an MHC protein complex.	57h18.2, abcb3l1, erap1a, tapbp.1, mhc1uka, erap1b, ctss2.2, mhc2dab			

Differentially expressed in 'aged mutant vs. young mutant' (1,072 genes)

Enriched functional term	Description	Gene Names	% Genes with term	% Background genes with term	Bonferroni *p*-value
Homeobox Antennapedia CS (IPR001827, *Interpro domains*)	Antennapedia subfamily of homeobox genes	hoxb5a, hoxa5a, hoxc4a, hoxb6b, hoxb6a, hoxa4a, hoxb8a, hoxc5a, hoxb3a, hoxc6a, hoxb5b, hoxb8b	1.43%	0.12%	2.78E-07
Circadian regulation of gene expression (GO:0032922, *GO Biological Process*)	Any process that modulates the frequency, rate or extent of gene expression such that an expression pattern recurs with a regularity of approximately 24 hours.	nr1d2a, nr1d1, ciarta, per3, nfil3-5, bhlhe40, clocka	1.00%	0.08%	0.00370
GSE21546 WT VS SAP1A KO DP THYMOCYTES UP (*MSigDB*)	Genes up-regulated in untreated double positive thymocytes: wild type versus ELK4 knockout.	psme2, b2ml, zgc:171731, b2m, dhx58, usp18, eif2ak2, mhc1uka, tjp1a, mxc, psmb9a, tapbpl, psmb8a, xaf1, nrg2a, csnk1g1, csrnp1b, vezf1b, abcb3l1, mhc1uba, pfkfb3, si:dkey-57h18.2, arsh	3.67%	1.05%	0.00421

Age-dependent inversion between mutant and wild type brains (65 genes)
(opposite direction of differential expression in 'young mutant vs. young wild type' and 'aged mutant vs. aged wild type'

Enriched functional term	Description	Gene Names	% Genes with term	% Background genes with term	Bonferroni *p*-value
CHEN LVAD SUPPORT OF FAILING HEART UP (*MSigDB*)	Genes up-regulated in left ventrical myocardium of patients with heart failure	pik3r1, ddit4, gadd45ba, nfkbiab, irs2b, fkbp5, mcl1b	21.21%	0.73%	0.000125
GSE14769 UNSTIM VS 40MIN LPS BMDM DN (*MSigDB*)	Genes down-regulated in comparison of unstimulated macrophage cells versus macrophage cells stimulated with LPS (TLR4 agonist) for 40 min.	LOC566587, stk35, tob1a, mcl1b, ifrd1, nfkbiab, gadd45ba, csrnp1b	24.24%	1.25 %	0.000224
GSE46606 UNSTIM VS CD40L IL2 IL5 1DAY STIMULATED IRF4HIGH SORTED BCELL DN (*MSigDB*)	Genes down-regulated in at day 0 B cell IRF4-KO versus CD40L and IL-2 IL-4 IL-5 stimulated at day 1 B cell IRF4high.	LOC566587, ifrd1, irs2b, mcl1b, tagapb, csrnp1b, nfkbiab	21.21%	1.14%	0.00299

Accelerated aging (65 genes)
(same direction of differential expression in 'young mutant vs. young wild type' and 'aged wild type vs. young wild type')

Enriched functional term	Description	Gene Names	% Genes with term	% Background genes with term	Bonferroni *p*-value
GSE9988 LPS VS VEHICLE TREATED MONOCYTE UP (*MSigDB*)	Genes up-regulated in comparison of monocytes treated with 1 ng/ml LPS (TLR4 agonist) versus untreated monocytes.	plk3, ddit4, gadd45ba, nfkbiab, csrnp1b, tnfaip2a, adrb2b	21.88%	1.00%	0.000948

Inappropriately downregulated (57 genes)
(down-regulated in both 'aged mutant vs. young mutant' and 'aged mutant vs. aged wild type')

Enriched functional term	Description	Gene names	% Genes with term	% Background genes with term	Bonferroni *p*-value
Homeobox Antennapedia CS (IPR001827, *Interpro domains*)	Antennapedia subfamily of homeobox genes	hoxc5a, hoxa4a, hoxb3a, hoxb8a, hoxb5b, hoxa5a, hoxb5a	13.46%	0.12%	4.25E-09
CHEN LVAD SUPPORT OF FAILING HEART UP (*MSigDB*)	Genes upregulated in left myocardium after heart failure	gadd45ba, fkbp5, rnd3a, pik3r1, foxo3b, nfkbiab, ddit4, mcl1b	27.59%	0.73%	8.55E-07
Regulation of cellular metabolic process (GO:0031323, *GO Biological Process*)	Any process that modulates the frequency, rate or extent of the chemical reactions and pathways by which individual cells transform chemical substances.	nr1d2a, foxo3b, sart3, hoxa5a, hoxc1a, tnfrsf11a, hoxb5b, tsc22d3, phox2bb, si:ch73-380l10.2, hoxb5a, hoxb8a, gadd45ba, mcl1b, nfil3-5, rgcc, arntl1b, pik3r1, hoxb3a, tob1a, hoxa4a, hoxc5a, pik3r3a	47.92%	15.45%	0.00504

Failure to upregulate (94 genes)<br>(up-regulated in ‘aged wild type vs. young wild type’ and not up-regulated in ‘aged mutant vs. aged wild type’)					
**Enriched functional term**	**Description**	**Gene Names**	**% Genes with term**	**% Background genes with term**	**Bonferroni *p*-value**
GSE339 EX VIVO VS IN CULTURE CD8POS DC DN (*MSigDB*)	Genes down-regulated in comparison of ex vivo CD8 dendritic cells versus cultured CD8 DCs.	tiparp, kpna1, nfkbiab, mapk6, pik3r1, ulk2, gadd45ba, mxi1, Ifrd1	16.98%	1.51%	0.00344
GSE46606 UNSTIM VS CD40L IL2 IL5 1DAY STIMULATED IRF4HIGH SORTED BCELL DN (*MSigDB*)	Genes down-regulated in at day 0 B cell IRF4-KO versus CD40L and IL-2 IL-4 IL-5 stimulated at day 1 B cell IRF4high.	nfkbiab, irs2b, dnajb4, tagapb, ifrd1, LOC566587, stk40, csrnp1b	15.09%	1.14%	0.00556
GSE14769 UNSTIM VS 40MIN LPS BMDM DN	Genes down-regulated in comparison of unstimulated macrophage cells versus macrophage cells stimulated with LPS (TLR4 agonist) for 40 min.	nfkbiab, tiparp, stk35, dnajb4, LOC566587, csrnp1b, gadd45ba, ifrd1	15.09%	1.25%	0.0116

Aging signature (525 genes)<br>(same direction of differential expression in ‘aged wild type vs. young wild type’ and ‘aged mutant vs. young mutant’)					
**Enriched functional term**	**Description**	**Gene Names**	**% Genes with term**	**% Background genes with term**	**Bonferroni *p*-value**
GO ANTIGEN PROCESSING AND PRESENTATION OF ENDOGENOUS PEPTIDE ANTIGEN (*MSigDB*)	The process in which an antigen-presenting cell expresses a peptide antigen of endogenous origin on its cell surface in association with an MHC protein complex.	b2ml, abcb3l1, mhc1zba, si:dkey-57h18.2, mhc1uba, mhc1uka, b2m, tapbp.1	2.80%	0.14%	5.87E-05
GSE37533 PPARG1 FOXP3 VS FOXP3 TRANSDUCED CD4 TCELL PIOGLITAZONE TREATED UP (*MSigDB*)	Genes up-regulated in CD4 T cells treated with pioglitazone and over-expressing: FOXP3 and PPARg1 isoform of PPARG versus FOXP3.	eif2ak2, vezf1b, mhc1uka, b2m, nfyba, lgals9l1, mhc1uba, usp25, b2ml, zgc:171731, psmb9a, xaf1, ctnnd2b, mef2cb, ctnnd2a, scamp1, cyp2p7, mxc, dhx58, psmb8a	6.99%	1.37%	8.58E-05
GSE21546 WT VS SAP1A KO DP THYMOCYTES UP (*MsigDB*)	Genes up-regulated in untreated double positive thymocytes: wild type versus ELK4 knockout.	mhc1uka, b2m, vezf1b, eif2ak2, b2ml, abcb3l1, pfkfb3, mhc1uba, xaf1, zgc:171731, psmb9a, arsh, psmb8a, csnk1g1, dhx58, si:dkey-57h18.2, mxc	5.94%	1.05%	0.000257
