# Supplementary material for: Accelerated brain aging towards transcriptional inversion in a zebrafish model of the K115fs mutation of human PSEN2
Source: PLoS One. 2020 Jan 24;15(1):e0227258. doi: 10.1371/journal.pone.0227258 (PMC6980398; doi:10.1371/journal.pone.0227258)
Supplement: S5 Table — (PDF) [file pone.0227258.s006.pdf]

Supplementary Table 5. Summary of enrichment of known zebrafish promoter motifs in sets of genes identified from differential gene expression analysis. Significantly enriched motifs with Bonferroni-adjusted *p*-value < 0.05 are included in this table. Background genes are the 18,296 genes used in the differential gene expression analysis.

Genes Analysed	Number	Enriched Motif Name	Bonferroni *p*-value	% Genes with motif	% Background genes with motif
**Differentially expressed in 'young mutant vs. young wild type'**	105	–	–	–	–
Down-regulated only	40	–	–	–	–
Up-regulated only	65	–	–	–	–
**Differentially expressed in 'aged mutant vs. aged wild type'**	177	GRE	0.005725	17.65%	4.86%
Down-regulated only	139	GRE	6.736e-04	18.89%	4.88%
Up-regulated only	38	–	–	–	–
**Differentially expressed in 'aged wild type vs. young wild type'**	1,795	ISRE	3.633e-05	7.77%	3.92%
		IRF8	1.346e-04	21.81%	15.52%
		IRF1	2.044e-03	12.65%	8.29%
		PU.1:IRF8	3.336e-03	11.06%	7.08%
		IRF2	5.285e-03	10.36%	6.59%
Down-regulated only	692	–	–	–	–
Up-regulated only	1,103	ISRE	7.634e-06	9.16%	3.96%
		IRF8	2.509e-05	24.01%	15.6%
		PU.1:IRF8	4.747e-04	12.64%	7.10%
		IRF2	1.576e-03	11.69%	6.62%
		IRF1	4.879e-03	13.59%	8.36%
		Atf3	6.019e-03	36.65%	28.69%
		Fra2	0.03700	25.12%	18.86%
		Ets-distal	0.04120	15.01%	10.12%
		AP-1	0.04818	36.97%	29.9%
**Differentially expressed in 'aged mutant vs. young mutant'**	1,072	–	–	–	–
Down-regulated only	378	–	–	–	–
Up-regulated only	694	–	–	–	–
**Age-dependent inversion between mutant and wild type brains** (opposite direction of change in 'young mutant vs. young wild type' and 'aged mutant vs. aged wild type')	65	GRE	0.004735	23.91%	4.97%
**Accelerated aging** (same direction of differential expression in 'young mutant vs. young wild type' and 'aged wild type vs. young wild type')	65	–	–	–	–
**Inappropriately down-regulated** (down-regulated in both 'aged mutant vs. young mutant' and 'aged mutant vs. aged wild type')	57	GRE	0.000105	28.26%	4.99%
		ARE	0.0344	26.09%	7.34%
**Failure to up-regulate** (up-regulated in 'aged wild type vs. young wild type' and not up-regulated in 'aged mutant vs. aged wild type')	94	–	–	–	–
**Failure to downregulate** (down-regulated in 'aged wild type vs. young wild type' and not down-regulated in 'aged mutant vs. aged wild type')	26	–	–	–	–
**Aging signature** (same direction of differential expression in 'aged wild type vs. young wild type' and 'aged mutant vs. young mutant')	525	–	–	–	–
